# Tight Junction barriers in human hair follicles – role of claudin-1

**DOI:** 10.1038/s41598-018-30341-9

**Published:** 2018-08-24

**Authors:** Michaela Zorn-Kruppa, Sabine Vidal-y-Sy, Pia Houdek, Ewa Wladykowski, Stephan Grzybowski, Robert Gruber, Christian Gorzelanny, Jason Harcup, Stefan W. Schneider, Amitabha Majumdar, Johanna M. Brandner

**Affiliations:** 1 Department of Dermatology and Venerology, University Hospital Hamburg-Eppendorf, Hamburg, Germany; 2 Medical One Klinik, Hamburg, Germany; 3 Department of Dermatology, Medical University of Innsbruck, Innsbruck, Austria; 4 Unilever R&D Port Sunlight Laboratory, Bebington, UK; 5 Unilever R&D Bangalore, Bangalore, India

## Abstract

Barrier function of hair follicles (HFs) is of great interest because they might be an entry port for allergens/pathogens, but could on the other hand be used for drug delivery or vaccination. Therefore we investigated tight junction (TJ) barrier function in human HFs. We show that there is a TJ barrier in the outermost living layer bordering to the environment from the infundibulum to the lower central part and between Henle’s and Huxles layer of anagen HFs. In club hair typical for catagen and telogen HFs a TJ barrier is found surrounding the club. This demonstrates that there is a continuous TJ barrier along interfollicular epidermis and HFs in different phases of HF cycle. However, interestingly, in cell culture experiments we can show that barrier is less tight in HF keratinocytes compared to interfollicular keratinocytes. Knock-down of the TJ protein claudin-1, which we demonstrate here to be less expressed in HFs of lesional atopic dermatitis skin, results in impaired barrier function, decreased proliferation and increased apoptosis of hair keratinocytes. This is in line with a hair growth phenotype in claudin-1 deficient patients (NISCH syndrome) and corresponding knock-out mice and indicates an important role of claudin-1 in HF barrier function and growth.

## Introduction

Human hair follicles (HFs) can be found throughout the surface of our body except for glabrous skin. The HF is a very complex structure consisting of infundibulum, isthmus, central region, suprabulbar region and bulb. When looking at horizontal cross sections of the HF, the hair shaft is wrapped by the inner root sheath (IRS) and the outer root sheath (ORS)^1^ (for a more detailed description see Supplementary Fig. S1).

The HF undergoes consecutive cycles of growth, regression and resting periods, namely anagen, catagen and telogen. During hair cycle the lower portion of the HF is renewed while the upper portion is the permanent part which is in continuity with the epidermis. Anagen is the rapid growth phase and is characterized by increasing length of the HF and the hair fibre. During catagen which is the regressing phase, the length of the HF decreases and the epithelial compartment of the lower follicle, including the matrix cells and the differentiated layers, undergoes apoptosis. In contrast, the dermal papilla (DP), although it shrinks, remains intact. The anagen hair shaft is now transformed to a club hair. Between club hair and DP the epithelial strand is found^2^. In telogen, which is the resting phase, the follicle is situated in the dermis with a small finger of epithelial cells above a cluster of DP cells. When the telogen follicle transits to the next anagen phase, the cells of the germ, which are at the base of the epithelial cells, swell and grow down to enclose the papilla. During early anagen, the club hair is lost while a new hair is formed. Coordinated proliferation and apoptosis are important for the hair cycle.

In recent years, HFs have gained interest as entry route for external substances which can be positive, e.g. in drug delivery, or negative, e.g. by uptake of pathogens, pollutants and allergens^3–7^. Therefore knowledge of the HF barrier function is of importance.

In the epidermis, two major structures are known to perform (mechanical) barrier function: the *stratum corneum* (SC) and tight junctions (TJs). TJs are complex cell-cell junctions in the *stratum granulosum* (SG)^8–10^. Epidermal TJs consist of a variety of TJ transmembrane proteins, namely claudin (Cldn)-1, Cldn-4, Cldn-7, occludin (Ocln) and junctional adhesion molecule-A (JAM-A), as well as TJ-plaque proteins such as *zonula occludens* (ZO)-1, ZO-2 and cingulin^8,10–14^. Several TJ proteins are not limited to barrier-forming TJ structures in the SG but are also found in other epidermal layers^14^.

In porcine anagen HFs, a continuous TJ barrier was demonstrated from the infundibulum down to the lower central/upper suprabulbar region. In the infundibulum the barrier was found in the SG. In isthmus, central region and upper suprabulbar region the TJ barrier was localized in the companion cell layer (CL) of the ORS^1^. Furthermore, an additional TJ barrier was observed between Henle’s and Huxley’s layer of the lower central/upper supbrabulbar region. In the bulbar region no barrier was found^1^.

However, TJ barriers in human HFs have not been investigated yet, even though presence and localization of various TJ proteins were described^11,15–17^, and TJ structures were shown on ultrastructural level in the CL^18^. Therefore the first aim of our project was to investigate presence and localization of TJ barriers in human anagen HFs which we can demonstrate to be indeed identical to porcine HFs. Because barrier function is not only important in anagen phase of the hair cycle, localization of TJ proteins and TJ barrier was also explored in other phases of HF cycle and we demonstrate that functional TJs are also present surrounding the club hair of catagen and telogen. In addition, up to now barrier permeability was only tested for a molecular tracer of 557 Da^1^. Thus, we further investigated barrier function to ions (measurement of transepithelial resistance (TER)) and larger tracer molecules (4 kDa fluorescein isothiocyanate-dextran (FD4)) which were measured in cultured anagen HF keratinocytes. We show that barriers to ions and tracer molecules are formed, however less tight than in interfollicular keratinocytes.

Finally we investigated the role of the TJ protein Cldn-1 in HF keratinocytes. We were especially interested in Cldn-1 because (1) it is the most widespread TJ protein in HFs, (2) it shows a dynamic change during hair cycle, (3) it is known to be involved in proliferation^19–21^ and apoptosis^22^, which are important for hair growth and regression, (4) Cldn-1 knock-out in mouse and human results in an aberrant hair phenotype^9,23^, and (5) Cldn-1 is altered in the epidermis of skin diseases, such as atopic dermatitis (AD)^20,24^ and psoriasis^25,26^. We show here that Cldn-1 is also down-regulated in HFs of lesional AD skin and that down-regulation of Cldn-1 in hair keratinocytes results in impaired barrier function, decreased proliferation and increased apoptosis.

## Results

### TJs in human anagen HFs form barriers

**Figure 1:**
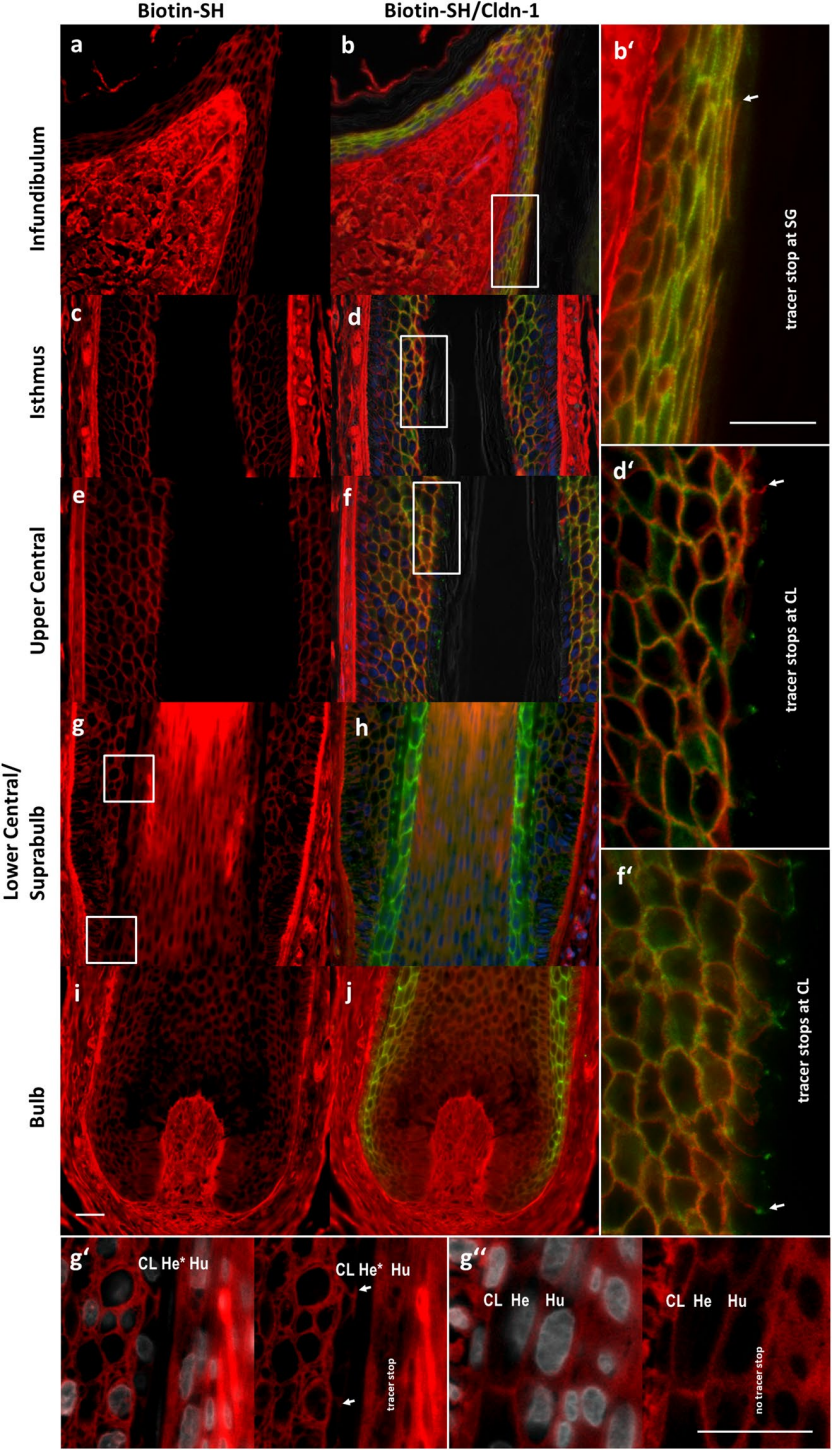
Localization of Cldn-1 and TJ barrier in human anagen HFs. Immunohistochemical stainings of Biotin-SH (red; all pictures) and Cldn-1 (green; **b**,b′,**d**,d′,**f**,f′,**h**,and **j** in an overlay with Biotin-SH) in epidermis and infundibulum (**a,b**,b′), isthmus region (**c,d,**d′), central/suprabulbar region (**e,f**,**g**,g′, g′′,**h**) and in bulbar region (**i**,**j**) of anagen HFs. (b′,d′ and f′) represent magnifications of (**b**,**d** and **f**) demonstrating localization of the tracer stop in the SG of the infundibulum and CL of isthmus and central region (arrows indicate exemplarily one stop in each picture). (g′ and g′′) represent magnifications of the boxed areas in **g** (g′: upper box; g′′: lower box), and are overlay images of Biotin-SH (red) and DAPI (white) (left pictures) or Bitoin-SH alone (right pictures) showing tracer stop at Huxley’s layer and at the CL of the ORS (arrows) above keratogenous zone at Adamson’s fringe with differentiated Henle’s layer cells (g′), whereas below no tracer stop was found (g′′). (CL: companion cell layer; He: Henle’s layer; He*: differentiated Henle’s layer; Hu: Huxley’s layer). Scale bars: 20 µm.

By using Biotin-SH which was injected into the dermis of human scalp skin (SSK) biopsies, we could observe TJ barrier function in the SG of the infundibulum (Fig. 1a,b,b′) and in the CL of the isthmus region (Fig. 1c,d,d′), of the upper central region (Fig. 1e,f,f′), and of the lower central/suprabulbar region (Fig. 1g,g′,h). In addition, a barrier was found at the border between Huxley’s and Henle’s layers in IRS (Fig. 1g,g′,h). Our data suggest that the barrier function and the structural assembly of human and porcine HFs are similar^1^.

**Figure 2:**
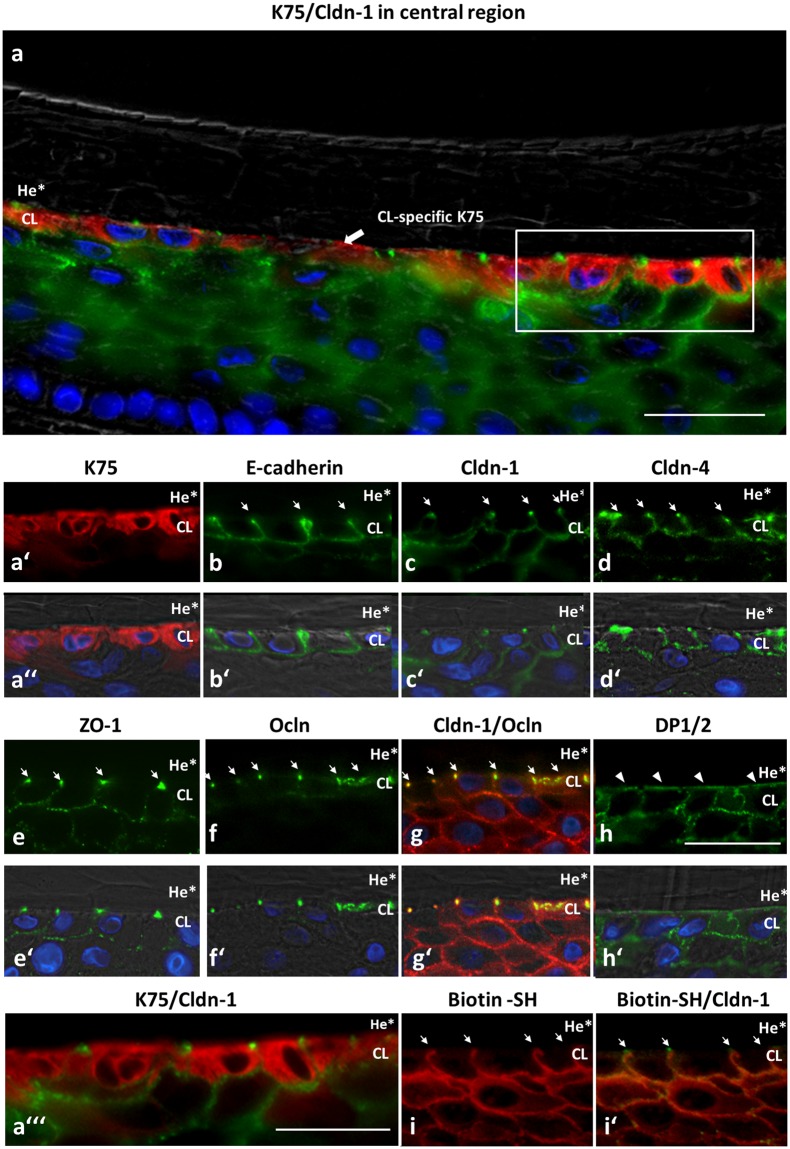
Identification of the CL via the marker protein K75 and demonstration of  CL polarization by distinct localization of several junctional proteins. Immunohistochemical stainings of K75 (red, **a**,a′,a′′,a′′′), Cldn-1 (green, **a**,a′′′,**c**,c′,i′ respectively red, **g**,g′), E-cadherin (green, **b**,b′), Cldn-4 (green, **d**,d′), ZO-1 (green, **e**,e′), Ocln (green, **f**,f′,**g**,g′), Desmoplakin (DP) 1/2 (green, **h**,h′) and Biotin-SH (red, **i**,i′) in the CL of the central region of anagen HFs. Note the polarized distribution of the junctional proteins and the stop of paracellular flux at functional TJs located at the apical sites of the lateral plasma membranes (arrows in b–g, i, i′) of the  CL bordering to the differentiated He* layer. Arrowheads in h denote the apical localization of DP 1/2. (**a**,a′′, b′–h′) Overlay images with DAPI staining (blue nuclei) as well as phase contrast. Scale bars: 20 μm.

In detail, we observed in the infundibulum - similar to the epidermis - a Biotin-SH tracer stop at the SG (Fig. 1a,b,b′), meaning that Biotin-SH was able to diffuse from the dermis into the living part of the ORS, yet got stopped at the SG of the ORS. This stop was found at the outermost sites positive for Cldn-1 (Fig. 1b,b′) which also denote in the epidermis the sites of functional TJs^9,27,28^. It is also the site of colocalization of Cldn-1 and Ocln (Supplementary Fig. S2). Also in the isthmus and central region, the Biotin-SH tracer was stopped at the last ORS layer positive for Cldn-1 (Fig. 1c–f′), which is here called the CL. Again, these sites were also positive for Ocln (Supplementary Fig. S2 for isthmus region). To verify that this site is the CL we performed stainings with CL specific K75 (Fig. 2a,a′,a′′,a′′′). Also in the lower central/suprabulbar region Biotin-SH diffused from the surrounding tissue into the ORS where it was stopped at the CL (Fig. 1g,g′,h).

In addition, the tracer was able to diffuse from the bulb upwards through matrix, hair cortex and cuticle, and stopped at the border between Huxley’s and Henle’s layers of IRS. Thus, diffusion of the tracer into the differentiated cells of Henle’s layer (He*) is stopped from both sides, the CL of the ORS and the Huxley’s layer, and makes these cells appear black (Fig. 1g,g′,h).

In areas with undifferentiated cells of Henle’s layer (He) this layer is accessible to the tracer (Fig. 1g,g′′,h). In the bulb no barrier was found (Fig. 1i,j).

Because these data reveal that the CL is equipped with a TJ barrier, we investigated whether this cell layer shows a polarized cell membrane as it has already been demonstrated for the SG of the interfollicular epidermis^29^. Indeed, we can show that this is the case. E-Cadherin, Cldn-1, Cldn-4 and ZO-1 are restricted to the basolateral plasma-membrane (Fig. 2b–e,b′–e′). The TJ proteins are more pronounced at the apical part of the lateral plasma membrane at sites of colocalization with Ocln which is restricted to this area (Fig. 2f,f′,g,g′). At these specific spots, Biotin-SH permeation is blocked (Fig. 2i,i′). On the other hand, Desmoplakins 1/2 are found basolateral and apical (Fig. 2h,h′).

To further confirm the role of TJs in barrier function of human HFs we investigated *ex vivo* HFs with and without EDTA to decrease extracellular Ca^2+^-concentration and open TJs prior to Biotin-SH treatment. Addition of EDTA resulted in significantly increased presence of Biotin-SH in the differentiated He*-layer revealing leaky TJs at the CL (Supplementary Fig. S3).

To confirm the *ex vivo* data for TJ barrier function of tracer molecules and to investigate HF-keratinocyte TJ barrier function to ions which is not easily possible in the skin samples, we subsequently isolated ORS keratinocytes from HFs and analyzed the TJ barrier *in vitro*.

**Figure 3:**
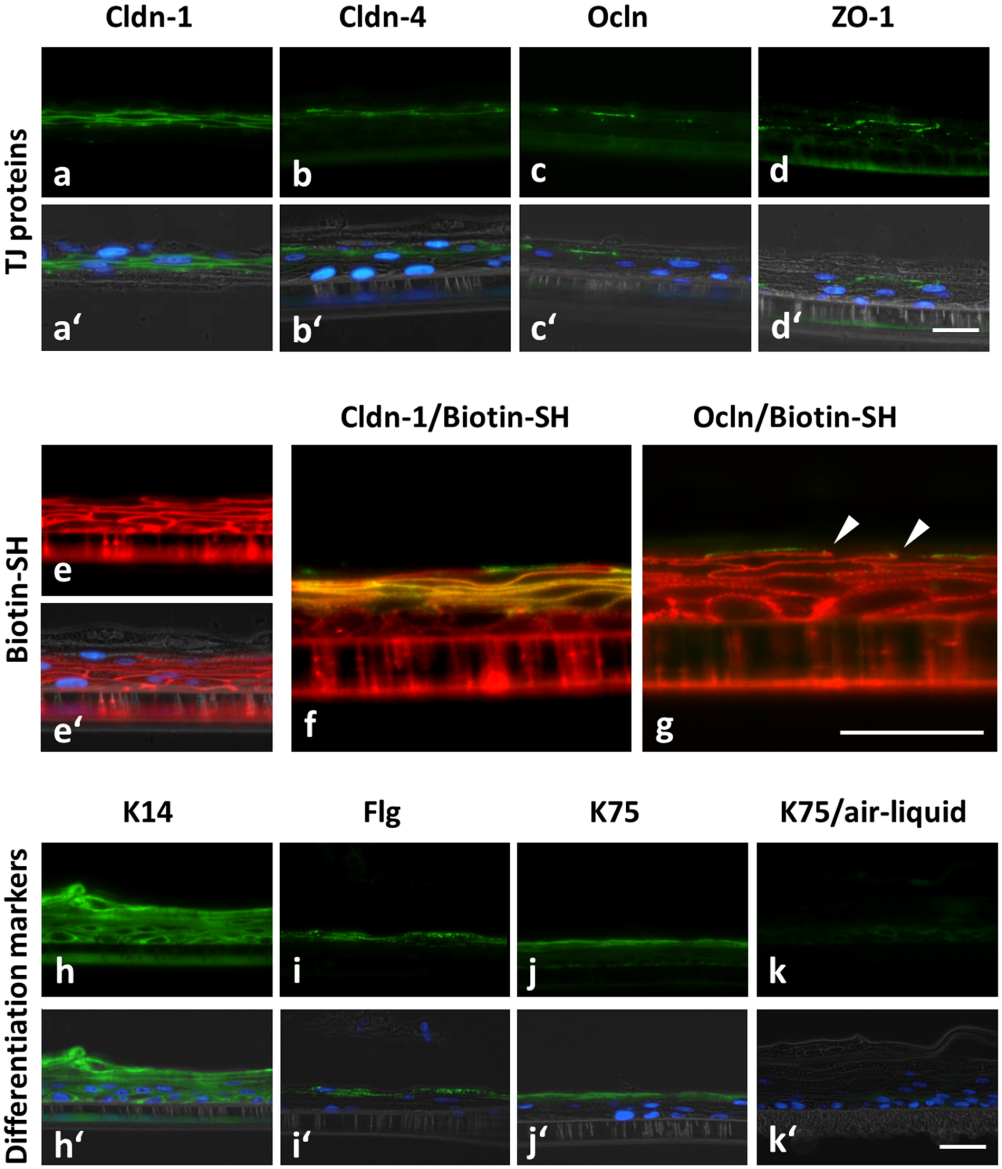
Localization of TJ proteins and TJ barrier as well as differentiation markers in cultured anagen HF keratinocytes. Immunohistochemical stainings of TJ proteins Cldn-1 (**a**,a′,**f**), Cldn-4 (**b**,b′), Ocln (**c**,c′,**g**), ZO-1 (**d**,d′) (green) and Biotin-SH (**e**,e′,**f**,**g**) (red) and differentiation marker proteins Keratin 14 (K14) (**h**,h′), Filaggrin (Flg) (**i**,i′), and Keratin 75 (K75) (**j**,j′) (green) in ORS keratinocytes isolated from human HFs and cultured submerged on Transwell filters for 8 days. (**k**,k′) K75 in ORS keratinocytes cultured under air-liquid-interface conditions at day 8. (a′,b′,c′,d′,e′,h′,i′,j′,k′) Overlay images with DAPI (nuclei; blue) and phase contrast pictures (grey). (**f**,**g**) Overlay images of Biotin-SH (red) and Cldn-1 (**f**) and Ocln (**g** arrows denote Biotin-SH stops at Ocln) (green) respectively. Scale bars: 20 µm. Note the HF-like localization of TJ proteins and differentiation markers in the submerged cultures and the absence of HF-specific K75 in air-liquid interface-cultures.

Isolated ORS cells were plated on Transwell filters and barrier functionality to Biotin-SH was checked after 8 days of submerged incubation under high calcium conditions (1.8 mM) when similar to the HF ORS a multilayered sheet was formed (for usage of submerged conditions for HF keratinocytes see^30^**)**. Comparable to the HF ORS, the cultured ORS keratinocytes showed a stratified structure and were positive for TJ proteins Cldn-1, Cldn-4, Ocln and ZO-1 in protein specific localization patterns: Cldn-1 was the most widespread and Ocln was restricted to the uppermost viable layer where all TJ proteins were present (Fig. 3a–d,a′–d′) and which was positive for K75, denoting a CL (Fig. 3j,j′; compare to Fig. 2 and^15^ for localization of TJ proteins in human anagen HF). Biotin-SH tracer was stopped at Cldn-1 and Ocln-positive sites, again similar to HF ORS, indicating a functional TJ barrier (Fig. 3e,e′,f,g). In addition, K14 showed HF-typical localization throughout all ORS layers (Fig. 3h,h′)^31^. The cultures were also positive for Filaggrin (Fig. 3i,i′), which is, however, only found in the infundibulum of ORS. Of note, when ORS cells were cultured in air-liquid-interface (ALI) they lost K75 staining in the uppermost layer (Fig. 3k,k′).

**Figure 4:**
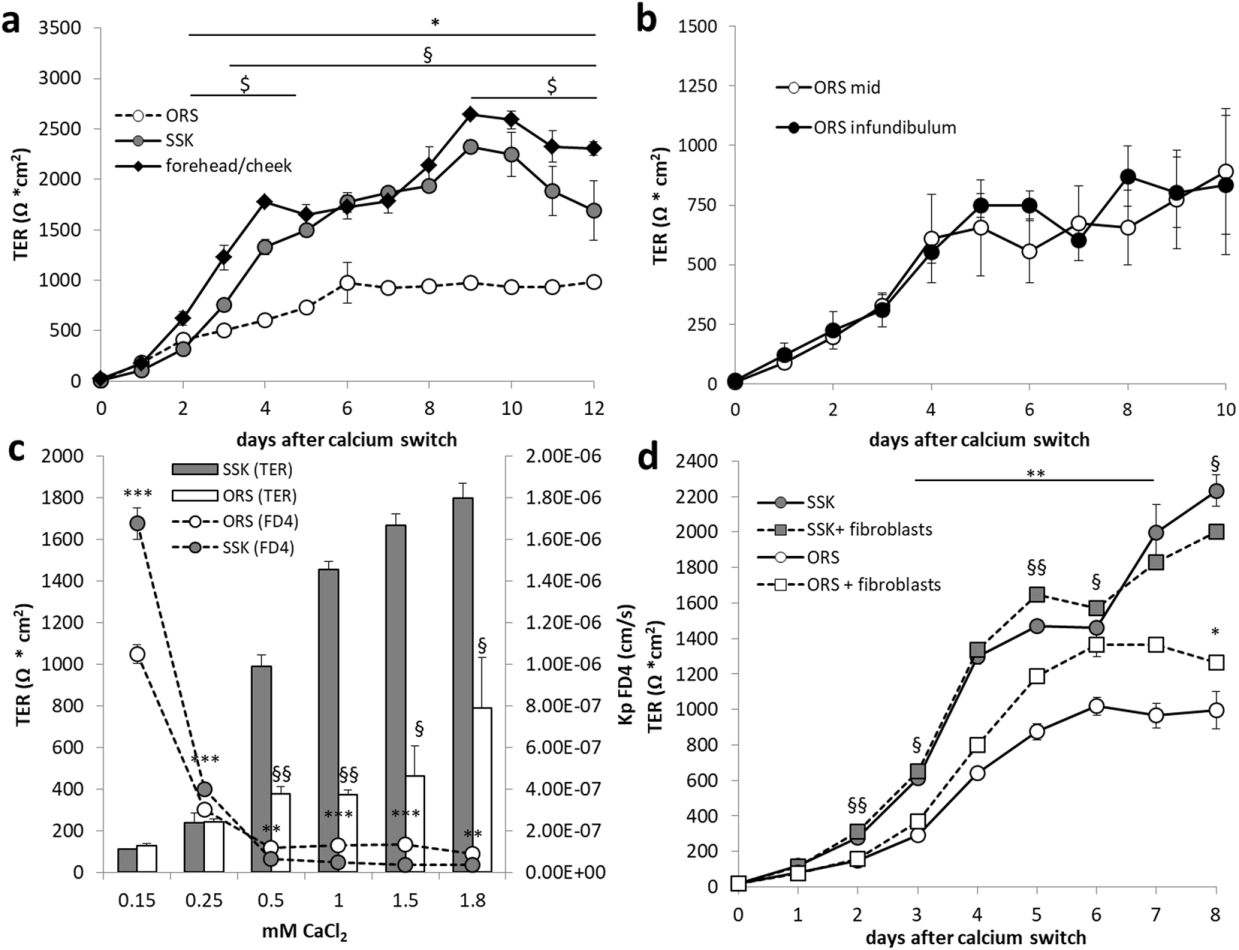
Barrier function of cultured HF keratinocytes. (**a**) Representative time dependent TER curve of keratinocyte cultures isolated from ORS, SSK, and forehead/cheek under submerged culture conditions. Means ± SD; triplicate wells n = 3 different donors; significant differences are indicated with * (between ORS and forehead/cheek) and § (between ORS and SSK) and $ (SSK and forehead/cheek), respectively. (**b**) Time dependent TER curve of ORS keratinocytes isolated from infundibulum (black circles), and mid segment (open circles) of HFs (means ± SEM; triplicate wells n = 3 different donors). (**c**) Representative TER diagram (column plot) in an overlay with the corresponding permeation coefficients for FD4 (line plot) of ORS and SSK keratinocytes at different calcium concentrations at day 8. Means ± SD for line plot or + SD for columns; triplicate wells, n = 4 different donors; significant differences are indicated by § for TER and by * for FD4 permeation. (**d**) Representative time dependent TER curve of ORS and SSK keratinocytes cultured in presence or absence of fibroblasts (means ± SD; triplicate wells, n = 3 different donors; significant differences between keratinocytes grown in presence and absence of fibroblasts are indicated by § for SSK and by * for ORS. Statistical test for all: ANOVA.

Keratinocytes isolated from ORS of anagen HFs formed a functional barrier to ions (Fig. 4). Formation started immediately after switch to high calcium and reached a first maximum after 4–6 days (Fig. 4a).

### Anagen HF keratinocyte barrier is less tight than scalp epidermis barrier and cheek barrier

When comparing keratinocytes isolated from anagen ORS, interfollicular SSK and forehead/cheek skin of the same donor, we found that forehead/cheek keratinocytes exhibited higher TER values than SSK keratinocytes and both TERs were significantly higher than those of ORS keratinocytes, indicating a weaker ORS barrier to ions (Fig. 4a). These significant differences were reproducible over 2 passages after isolation of cells (data not shown).

To clarify whether at least keratinocytes from the infundibulum, where structure of the cell sheets is more similar to the epidermis, form a higher TER we compared cells isolated from the infundibulum with those from the mid segment. However, both showed similar TER values (Fig. 4b), which were lower than those observed for interfollicular skin (Fig. 4a).

Because interfollicular keratinocytes are often cultured under ALI conditions, we compared barrier function of ORS and SSK cells also under these conditions which are optimized for SSK 3D models^32–34^. Also under these conditions we observed higher permeability to ions, i.e. lower TER, in ORS keratinocytes compared to SSK keratinocytes, even though not yet significant. When using Biotin-SH as a molecular tracer, we observed significantly higher permeability of ORS cells compared to SSK cells (Supplementary Fig. S4).

### Role of calcium and extracellular stimuli in barrier function of ORS and SSK keratinocytes

Formation of TJ barrier function depends on elevated extracellular Ca^2+^-concentration^35,36^. To elucidate the role of calcium in barrier formation of ORS and SSK keratinocytes, TER values of both cell types were recorded in response to different calcium concentrations ranging from 0.15 to 1.8 mM CaCl_2_. Interestingly, ORS and SSK cells responded differently to rising calcium levels regarding their barrier function, particularly at higher calcium concentrations (Fig. 4c). When comparing the TER values, it became obvious that at concentrations of 0.5 to 1.8 mM CaCl_2_ SSK showed a significantly tighter barrier whereas at concentrations below 0.5 mM CaCl_2_ TER of ORS and SSK cells were similar.

Complementary results were observed for the barrier permeation of the molecular tracer FD4. We observed increased permeability of ORS cells at higher Ca^2+^-concentrations compared to SSK cells. Of note, permeability of ORS cells was significantly lower than that of SSK cells at lower Ca^2+^-concentrations (Fig. 4c).

To investigate whether altered differentiation might be responsible for the differences observed in barrier function, we investigated localization and staining intensity of differentiation markers K14, K75 and Filaggrin at day 5 and day 8. However, neither at 0.5 mM nor at 1.8 mM CaCl_2_ a difference between ORS and SSK cells was detectable (Supplementary Fig. S5).

Because we observed a differential influence of extracellular Ca^2+^ on permeability of ORS versus SSK keratinocytes we wanted to know whether also other external stimuli, e.g. produced by the surrounding fibroblasts, might result in different behaviour of ORS and SSK. Therefore TER values of ORS keratinocytes were recorded during 8 days of cocultures with dermal fibroblasts isolated from SSK (Fig. 4d). Coculture was performed in such a way that the keratinocytes were cultured submerged on the transwell filters and the fibroblasts at the bottom of the wells. Remarkably, this experiment revealed that the fibroblasts improved the TER of ORS cultures from day 4–8. TER of SSK cells was less influenced by fibroblast cocultivation (Fig. 4d).

To exclude that a co-culture with fibroblasts at the beginning of cultivation directly after isolation might prevent the lower TER of ORS keratinocytes compared to SSK cells we cultured ORS keratinocytes directly after isolation with FAD/fibroblast-feeder layer. Also this cocultivation resulted in higher TER values in the first passage compared to the same cells initially cultured in DermaLife medium without fibroblast feeder layer (Supplementary Fig. S6). However, the values were still lower than for SSK keratinocytes (Fig. 4a).

### TJ proteins during HF cycle

During cycling, the HF undergoes dramatic anatomical changes. These changes include the formation of a club hair above the keratogenius zone in catagen which is still present in telogen and shed in exogen phase as well as the formation of an epithelial strand by remnants of the ORS and matrix cells (see simplified cartoon in Supplementary Fig. S7). The IRS disintegrates during regression. We wanted to know whether HFs also provide a barrier during cycling, and, because TJ proteins are known to be involved in proliferation and apoptosis, specify the fate of TJ proteins in human scalp HF during HF regression towards club hair formation.

**Figure 5:**
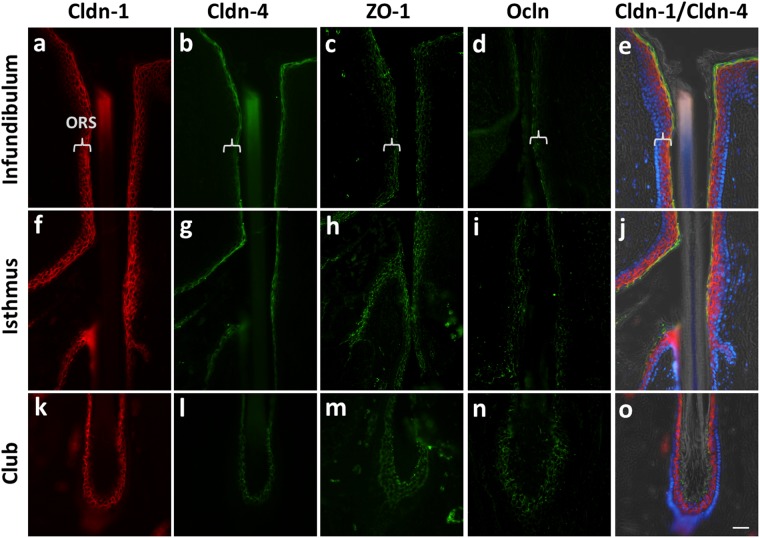
Localization of TJ proteins in human club hair follicles. Immunohistochemical stainings of Cldn-1 (**a,e,f,j,k,o**; red), Cldn-4 (**b,e,g,j,l,o**; green), ZO-1 (**c,h,m**; green), and Ocln (**d,i,n;** green). (**e,j,o**) Overlay of Cldn-1 and Cldn-4 double staining with DAPI (blue nuclei) and phase contrast. Parentheses denote ORS. Scale bar: 50 µm.

Concerning the upper, permanent, part, which is in continuity with the interfollicular epidermis, Cldn-1 was found in all viable layers of the infundibulum of the club HF (Fig. 5a,e), similar to anagen HFs (Fig. 1b). Intense staining for Cldn-4 was found in SG and SS (Fig. 5b,e) (for comparison to anagen HF see^1,15^). Concerning ZO-1 a weak staining was found in the upper epithelial layers and intense staining in the SG (Fig. 5c). Ocln was restricted to the SG (Fig. 5d) and thus again similar to the anagen HF.

Also concordantly to anagen HFs^1,15^, in the isthmus of club HFs Cldn-1 and ZO-1 staining were found in all living layers of ORS whereas Cldn-4 was only found in the outermost cell layers (Fig. 5f,g,h,j). However, contrary to Ocln staining in anagen isthmus where it is restricted to the CL, in the isthmus of the club HF it was found in the outermost  and also in central ORS layers in a sometimes patchy manner (Fig. 5i).

Since the club hair is a temporary structure formed during catagen which has to be split off from the body’s circulation at a certain time point and is finally shed after telogen phase, we were particularly interested in the TJ expression and localization pattern of cell layers surrounding this structure. Cldn-1 was clearly present in all layers of the ORS-derived, surrounding epithelia with the exception of basal cells (Fig. 5k,o). Cldn-4 staining was only found in the outermost layer of the epithelial tissue which is in direct contact to the brush-like keratinous rootlets of the club hair shaft (Fig. 5l,o). There it was found to be colocalized with Cldn-1 (Fig. 5o and Supplementary Fig. S8)

Similar to that, also Ocln was found in proximity to the keratinous rootlets at the outer layers of the epithelial sac surrounding the club (Fig. 5n) although it was stained in a broader range within 3–4 cell layers enclosing the brush. ZO-1 was positive in most of the ORS cell layers of the epithelial tissue enclosing the club (Fig. 5m) and both, Ocln and ZO-1 revealed colocalization with Cldn-1 in the last living layers which are in direct contact with the keratinous club (Supplementary Figs S9, S10).

In summary, when looking at TJ protein localization patterns in the infundibulum and isthmus of club HFs and in ORS surrounding the club hair shaft, we found colocalization of all tested TJ proteins in the outermost living epithelial layer bordering to the environment/club hair.

**Figure 6:**
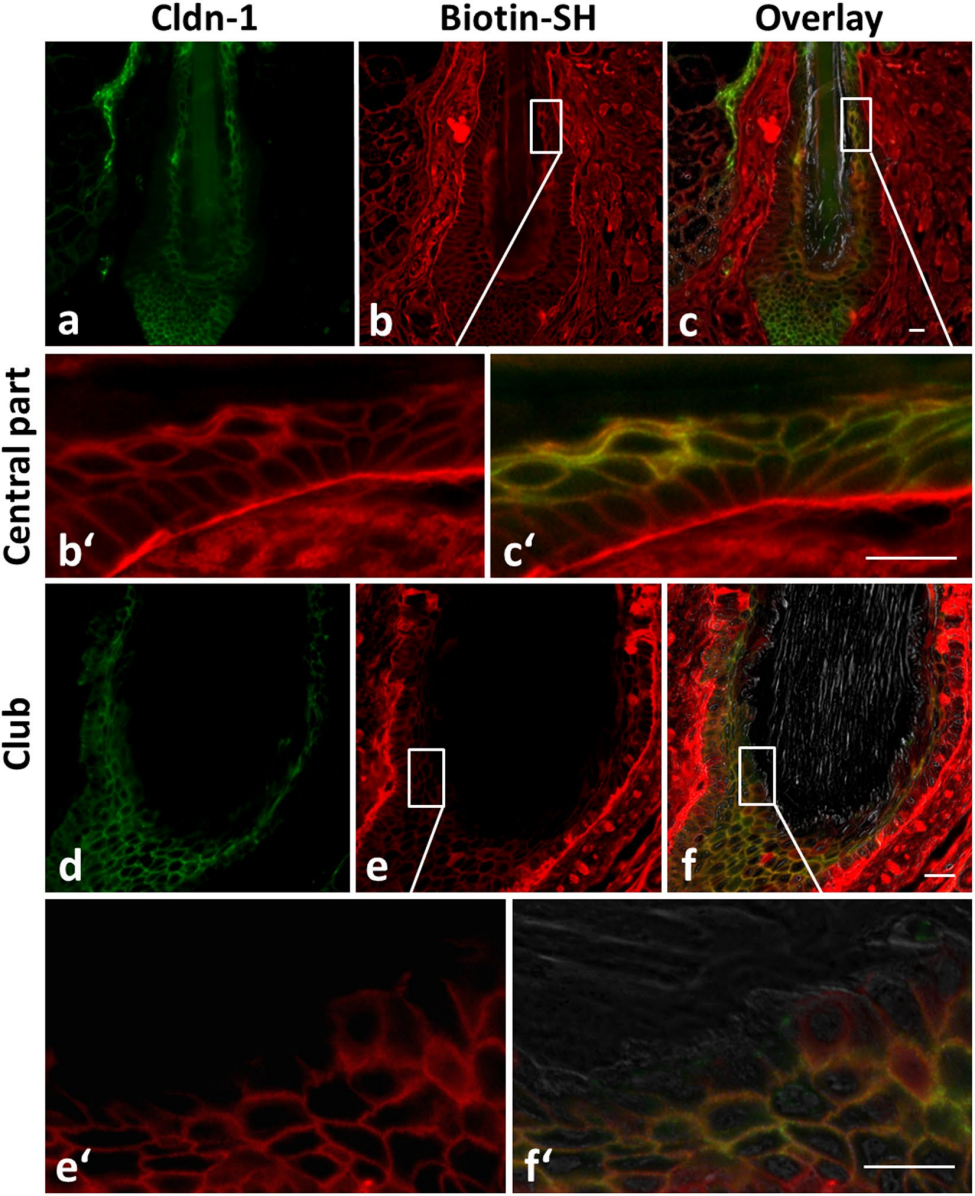
Localization of Cldn-1 and TJ barrier in human club hair follicles. Immunohistochemical stainings of Cldn-1 (green; **a,c**,c′,**d**,**f**,f′) and Biotin-SH (red; **b**,b′,**c**,c′,**e**,e′,**f**,f′) in the central part and around the brush of a club hair. (**c,c**′,**f**,f′) overlay of Cldn-1, Biotin-SH and phase contrast. (b′,c′,e′ and f′) represent magnifications of (**b**,**c**,**e**, and **f**) (which are rotated for 90°) showing that Biotin-SH permeation stops at the keratinized club. Scale bars: 20 µm.

Regarding TJ functionality, Biotin-SH stops were found in club HFs from the infundibulum down to the central region in the outermost living layer. In addition, functional TJs were also found in the outermost layers of the ORS surrounding the club hair in close proximity to the keratinous rootlets (Fig. 6).

Our studies thereby demonstrate that functional TJs form an effective seal between the hair and the dermis all around the keratinized club hair.

**Figure 7:**
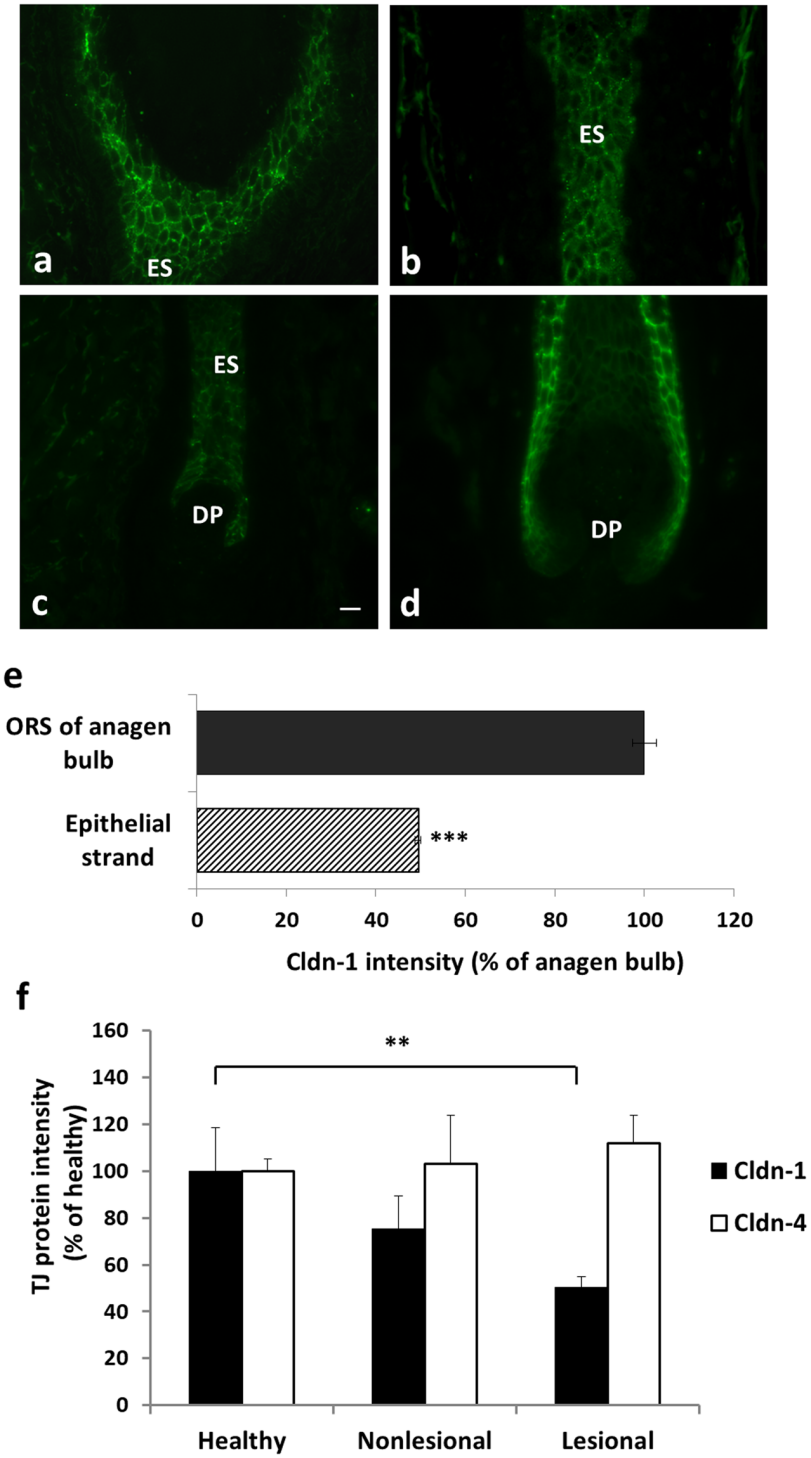
Quantification of Cldn-1 and Cldn-4 in human HFs. Immunohistochemical stainings of Cldn-1 (green; **a**–**d**) in the epithelium around the club hair (**a**), the epithelial strand (ES) (**a**,**b**,**c**) of club HF, and in the bulb of an anagen HF (**d**). Scale bar: 20 µm. (**e**) Quantitative analysis of Cldn-1 intensity in ORS of anagen bulb and epithelial strand during catagen (mean of 23 ROIs ± SEM, n = 3 different donors); (**f**) Cldn-1 and Cldn-4 staining intensity in human anagen HFs of healthy and AD non-lesional and lesional skin; means ± SEM of 10 ROIs per image. Statistical test: ANOVA, significant differences are indicated by*.

When looking at the cycling part which is localized below the club hair portion facing the dermal papilla during regression, we found that Cldn-1 was the only TJ protein significantly present in the epithelial strand (ES) (Fig. 7a–c), while Ocln, Cldn-4 and ZO1 were absent (data not shown). However, compared to anagen (Fig. 7d) also Cldn-1 was down-regulated (Fig. 7e). Moreover, subcellular localization of Cldn-1 changed: Staining pattern was less defined membranous and in a patchier manner compared to the epithelium of the anagen bulb (compare Fig. 7a–d).

### Cldn-1 and Cldn-4 in HFs in atopic dermatitis

Down-regulation of Cldn-1 in lesional epidermis of AD likely contributes to the vicious circle of AD by contributing to epidermal barrier impairment (for review see^37^). Because we have shown here that TJs also form a barrier in human HFs and therefore contribute to overall barrier function of the skin we wanted to check whether Cldn-1 is also affected in HFs of lesional or non-lesional skin from AD patients compared to healthy control skin. In addition, it was recently shown that Cldn-1 is also down-regulated in sweat glands of lesional AD skin^38^.

Indeed, we observed that Cldn-1 was also significantly down-regulated in HFs in lesional but not in non-lesional skin of AD patients compared to healthy controls (Fig. 7f). Of note, there was no difference in Cldn-4 levels in the HFs, even though there was an upregulation in non-lesional AD epidermis^24^ (Fig. 7f).

### Role of Cldn-1 in HF barrier function, proliferation and apoptosis

Patients without Cldn-1 (NISCH syndrome)^23^ and Cldn-1 knock-out mice^9^ show an altered hair phenotype. In addition, we show here that Cldn-1 is substantially down-regulated in the lower cycling part of club hairs and that it is down-regulated in HFs of lesional AD skin. Thus, we were interested in the specific role of Cldn-1 in HF keratinocytes. Therefore knock-down (KD) experiments using specific siRNAs were performed in ORS keratinocytes. Cldn-1 was down-regulated to 32.5% ± 14.6% by using Cldn-1-siRNA_5 and to 9.6% ± 5.9% by using Cldn-1-siRNA_8 at day 4 after calcium switch (respectively day 5 after KD).

**Figure 8:**
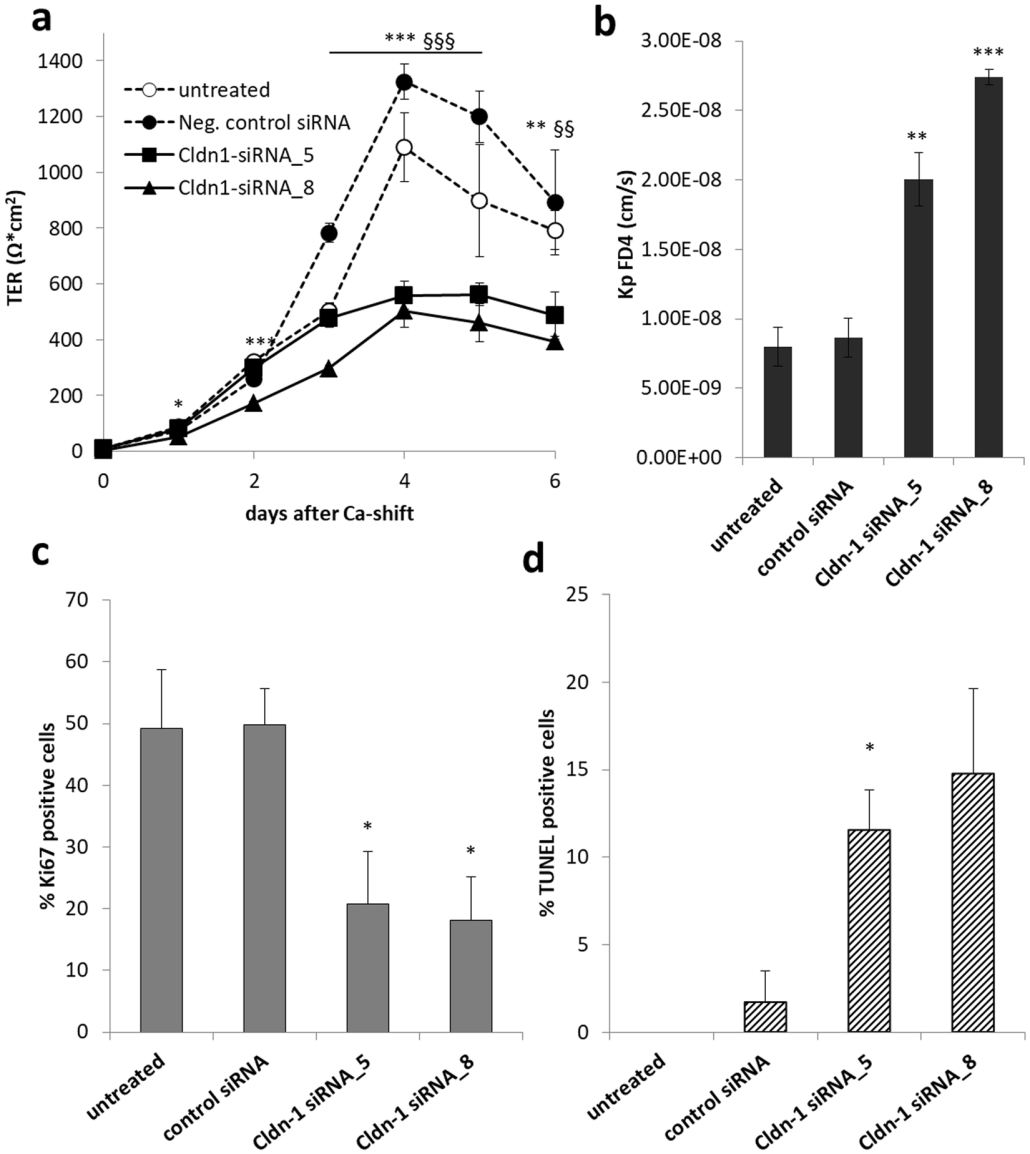
Effect of Cldn-1 KD on barrier function, proliferation and apoptosis in ORS keratinocytes. Representative diagrams showing barrier function to ions (TER, **a**) and FD4 (**b**) and percentage of proliferative (Ki67-positive, **c**) and apoptotic (TUNEL positive, **d**) cells after siRNA mediated KD of Cldn-1 using two different siRNAs (Cldn-1siRNA_5 and _8) and negative control siRNA in cultured ORS keratinocytes. (**a**) TER, means ± SD of 4 wells, n = 3 different donors; significant differences between negative control siRNA cells and cells incubated with siRNA_5 and Cldn-1siRNA_8 are indicated by § and *, respectively. (**b**) Coefficients for FD4 permeations (Kp) at day 4; means + SD of triplicate measurements, n = 3 different donors; *denotes significant differences between Cldn-1 siRNAs and control siRNA. (**c**) Proportion of proliferative (Ki67-positive) cells at day 2 after Ca^2+^-switch expressed as percentage of DAPI-positive cells, (**d**) Proportion of apoptotic (TUNEL-positive) cells at day 4 after Ca^2+^-switch expressed as percentage of DAPI-positive cells. (**c,d**) Means + SD of triplicate measurements, n = 3 different donors; significant differences between cells treated with control siRNA and those treated with either Cldn-1 sIRNA_5 or Cldn-1 sIRNA_8 are indicated by*. Statistical test for all experiments: ANOVA.

KD of Cldn-1 resulted in a significant decrease in ion barrier function (TER, Fig. 8a), and increased permeability for FD4 (Fig. 8b) with both siRNAs. The effect was more pronounced with Cldn-1 siRNA_8 (Fig. 8a,b) in correlation with a more pronounced KD. There was no effect on Ocln, E-Cadherin and Desmoplakin 1/2 and only a slight but non-significant increasing effect on Cldn-4 levels with siRNA_8 (Supplementary Fig. S11).

In addition, Cldn-1 KD resulted in significantly decreased proliferation of ORS keratinocytes (Fig. 8c). This was also confirmed by a decreased number of KD cells compared to control cells measured by MTT assay (data not shown). Moreover, the incidence of apoptotic cells (TUNEL positive cells) was increased by Cldn-1 KD (Fig. 8d). At day 8, when Cldn-1 expression levels returned to normal (85% ± 18%) also the degree of proliferation and apoptosis were at the same levels as the controls (Ki67: 22.1% ± 4.2% for Cldn-1-siRNA_5, 28.8% ± 2% for Cldn-1-siRNA_8, 27.7% ± 2.9% for control siRNA; TUNEL: 17% ± 6.2% for Cldn-1-siRNA_5, 18.1% ± 5.7% for Cldn-1-siRNA_8, 13.1% ± 3% for control siRNA ( ± SEM).

## Discussion

It was shown previously that TJs form a functional barrier in porcine anagen HFs^1^, but proof of a functional TJ barrier in human HFs was missing. We show here that, similar to porcine skin, a functional barrier is found in the outermost living layers of the human anagen HF bordering to the environment i.e. the SG of the infundibulum and the CL of the isthmus and central/suprabulbar region. Thus, there is a continuous TJ barrier along the epidermis and accessible areas of human anagen HFs. Furthermore, we demonstrate that the HF barrier can be impaired by opening TJs due to removal of extracellular calcium. In line with the presence of TJs in the CL, and also as an important prerequisite for functional TJs, we could show that this layer, similar to the SG in the epidermis^29,39^, is a polarized cell layer.

In the infundibulum, in addition to TJs, SC is present^40^, resulting in a double barrier in this area. This is, again, similar to porcine HF^1^ and substantiates that the porcine HF is indeed a good model to investigate human HF barrier function.

Investigation of the uptake of nanoparticles into porcine HFs showed that most of these nanoparticles were found in the area where two barriers, SC and TJs, are present, some in areas with one barrier (TJs), and none in areas without TJ barrier^1^. This data led to the conclusion that nature has made a kind of “risk stratification” with protecting the body by two barriers where it is easily accessible to environmental hazards, one barrier at sites with a minor risk of accessibility and no barrier where no hazard can reach the body. It is very likely that the same is true for human HFs as we find identical barrier structures in pig and human. Unfortunately it is not possible to perform *ex vivo* uptake experiments in human HFs because they collapse after excision of the skin^41^.

Also at the border between Huxley’s and differentiated Henle’s layer a TJ barrier was observed, leading to an isolation of the differentiated Henle’s layer because this layer is as well not accessible via the TJ barrier forming CL. One might suggest that this prevents the loss of precious nutrients, hormones and signaling molecules to this dead and subsequently shed off layer.

When looking at the HF at other stages of HF cycle, namely at the club hair of catagen and telogen phase, we found a functional barrier which completely surrounds the club hair, thus isolating the club hair from the surrounding dermis. Again, this may be of special importance to limit the loss of nutrients, hormones and cytokines into the club hair. In addition, it may contribute to the subsequent shedding of the club hair. Finally, this results in a continuous barrier along the interfollicular epidermis and HFs against the environment also during HF cycle.

Our data in submerged cultured ORS keratinocytes which grow as a stratified multilayered sheet with K75 expression in the uppermost viable layer and TJ protein distribution similar to *in vivo* ORS^15^ show that there clearly exists a barrier to ions (TER) and tracer molecules (FD4, Biotin-SH), but it is less tight compared to interfollicular scalp keratinocytes.

Of note, we detected no differences in differentiation markers between ORS and SSK cell cultures. However, there may be differentiation markers not tested here that might show differences. Thus, we cannot completely exclude that altered differentiation may contribute to the differences in permeability observed between cultured ORS and SSK cells.

We chose submerged conditions for our cultured keratinocytes because it was previously shown that these conditions are more suited for HF cells than ALI conditions due to the microenvironment in the HF^30^. However, because interfollicular keratinocytes are normally cultured in ALI^34^ we also performed permeability assays under ALI conditions. Nonetheless we still saw more permeability in ORS derived cells.

Our data also hint for a difference in TER between interfollicular SSK and cheek skin. It was shown before that permeation of coumarin and propranolol was significantly higher in SSK compared to abdominal skin^42^. However, because HFs are important permeation routes and are present in high amount in the SSK, it is not possible to clearly distinguish in these published results between permeability of interfollicular SSK compared to scalp HFs.

Interestingly, the inferiority of barrier function of ORS keratinocytes was only found at higher extracellular Ca^2+^-concentrations. At lower concentrations ORS keratinocytes show a similar or even stronger barrier than SSK keratinocytes. Of note, to our knowledge, exact Ca^2+^ concentrations in HFs in comparison to the epidermis are not known yet. Thus, culture conditions of cultured cells are always just an approximation and *in vitro* experiments cannot replace experiments in *ex vivo* or *in vivo* skin. However, a combination of different *in vitro* models might add valuable information from different points of view.

In general, our data show that ORS keratinocytes are much less dependent from extracellular Ca^2+^-concentrations than SSK keratinocytes. One might argue that greater independency of the barrier function from extracellular calcium concentrations is important for a dynamic structure like the HF, which faces several microenvironments and maybe different calcium concentrations during HF cycle. However, this still has to be clarified in the future, as well as the question, why nature may have provided the HF with a less tight barrier compared to the interfollicular epidermis.

Although HF keratinocytes are less dependent from calcium concentrations than interfollicular keratinocytes, they are more influenced by fibroblasts than SSK keratinocytes. Because there was no direct physical contact in our co-cultures between fibroblasts and keratinocytes, soluble factors appear to influence the barrier function. It was shown before that FGFR1/R2 knock-out mice show decreased TJ protein levels (including Cldn-1) and a progressive loss of HFs. In addition, immortalized keratinocytes from FGFR1/R2 mutant mice show decreased TER^43^. Treatment of mouse keratinocytes with FGF-7 resulted in increased Cldn-1 levels^43^.

Here, we demonstrated that Cldn-1 is the most abundant TJ protein in anagen as well as in club HFs. However, its expression decreases in catagen epithelial strand cells compared to anagen bulb cells. In addition, decreased expression was observed in HFs of lesional AD skin while there was no change in Cldn-4. Together with the knowledge of an aberrant HF phenotype in patients with NISCH syndrome and Cldn-1 knock-out mice this prompted us to investigate the role of Cldn-1 in HF keratinocytes.

We observed that KD of Cldn-1 resulted in impaired barrier function in ORS keratinocytes, similar to epidermal keratinocytes^9,36,44,45^. Thus, the down-regulation of Cldn-1 in HFs of lesional skin of AD might contribute to the impaired skin barrier function of patients with AD. Furthermore, we observed a decrease of proliferative cells in Cldn-1 KD keratinocytes. Previously it was shown that KD of Cldn-1 resulted in increased proliferation in epidermal keratinocytes^20^. In addition, a positive correlation between Cldn-1 expression and amount of proliferative cells in the epidermis was observed in an AD-like allergic dermatitis mouse model^24^. These data indicate different biological properties and functions of epidermal and HF keratinocytes. However, recently a decrease of proliferative cells in epidermal keratinocytes has also been observed after Cldn-1 KD in wounded epidermal keratinocyte monolayers^46^. This hints for an influence of microenvironment on the effect of Cldn-1 on proliferation.

In addition, we observed an upregulation of apoptotic cells in Cldn-1 KD cells. In line with our results it was previously shown that Cldn-1 KD results in an activation of the apoptosis-specific caspase-3 pathway in gastric cancer cells^47^ and in increased TNFα induced apoptosis in MCF-7 cells^48^. Cldn-1 overexpression resulted in reduced apoptosis in nasopharyngeal carcinoma^49^.

Thus, in summary we suggest that Cldn-1 expression in HFs is not only important for HF barrier function but also for hair growth and hair cycle progression. Future studies investigating HF disorders may contribute more information to this new aspect of Cldn-1 biology.

## Methods

### Tissues, antibodies, primers, siRNAs, chemicals

Human SSK samples from 15 female and 3 male adult donors (51+/−11 years) used for cell culture and IHC were obtained anonymously after medical interventions, most of them undergoing plastic surgery. The usage was approved by the ethics committee of the Aerztekammer Hamburg (OB-15/04, WF-28/12, WF-61/12). Skin samples from patients with AD and controls were previously described^24^. The study complied with the declaration of Helsinki principles. All subjects provided written informed consent. Because not all samples contained HFs we could evaluate 4 lesional, 7 non-lesional and 7 healthy samples. For antibodies see Supplementary Table S1. siRNA Hs_CLDN1_5 (Cldn-1 siRNA_5), siRNA Hs_CLDN1_8 (Cldn-1 siRNA_5) and negative control siRNA were all obtained from Qiagen (Hilden, Germany). FAM dye-labeled TaqMan MGB probes for Cldn-1 and GAPDH were obtained from Thermo Fisher Scientific (Darmstadt, Germany).

### Isolation and cultivation of ORS keratinocytes, interfollicular SSK keratinocytes, and forehead or cheek keratinocytes from human SSK biopsies

SSK biopsies were incubated overnight at 4 °C in RPMI medium (containing 10% fetal calf serum (FCS) and 1% penicillin/streptomycin). Then part of the subcutaneous tissue was carefully removed and biopsies were cut into dissectible pieces of 0.3 × 0.5 cm. The tissue fragments were put into dispase solution (2.5 mg/mL Dispase II (Roche, Mannheim, Germany)) and incubated at 37 °C for 4 h. Afterwards HFs were plucked out with very gentle force according to^50^. Only HFs in anagen stage were selected by stereo microscopic evaluation according to^2^ and then subsequently microdissected under a stereo loupe using a scalpel and thin tweezers. Thereafter, ORS cells were isolated by trypsinization (30 min, 0.25% trypsin, 37 °C). The cells were detached mechanically from hair keratin using a Pasteur pipette, neutralized by adding 3 volumes of 10% FCS and separated by centrifugation^51^. Cells from at least 10 HFs were plated in 2 mL medium in a 35 × 10 mm petri dish.

For isolation of interfollicular SSK or forehead/cheek keratinocytes, corresponding tissue regions of the SSK biopsies were cut into 2–3 mm^2^ pieces and kept in 0.25% trypsin overnight at 4 °C or 4 h at 37 °C. The epithelial layers were peeled off and collected in RPMI medium (containing 10% FCS and 1% penicillin/streptomycin). A single cell suspension was produced mechanically by repeated pipetting. Thereafter cells were centrifuged and plated in a density of 100,000 cells/mL in petri dishes. ORS, SSK and forehead/cheek keratinocytes were then cultured in serum-free keratinocyte growth medium (DermaLife, Lifeline/Cellsystems, Troisdorf, Germany).

For fibroblast isolation from SSK, the tissue biopsies were cut into <1 mm^2^ pieces and transferred to a 25 cm² culture flask with 2.5 mL RPMI medium (supplemented with 10% FCS, 1% glutamine and 1% penicillin/streptomycin). After 7–10 days outgrown fibroblasts were trypsinized and passaged before they were used for experiments. For coculture experiments with ORS and SSK keratinocytes, 40,000 keratinocytes were plated on Transwell filter inserts and 40,000 fibroblasts were plated beneath the inserts.

For certain experiments, the freshly isolated ORS cells were initially cultured (passage 0) according to a method modified after^52^ on (lethally irradiated) fibroblast feeder in complete FAD medium before cells were switched to DermaLife for further passages. FAD is made from DMEM/ Ham’s F12 (3:1) supplemented with 1.8 × 10^−4^ M adenine, 100 IU/mL penicillin, 100 µg/mL streptomycin, 10% fetal bovine serum, 0.5 ug/mL hydrocortisone, 8.47 ng/mL cholera enterotoxin, 10 ng/mL epidermal growth factor and 5 µg/mL insulin.

### siRNA experiments

ORS keratinocytes were transfected under low Ca^2+^-conditions (0.06 M) by using HiPerFect Transfection reagent (Qiagen, Hilden, Germany) according to the manufacturer’s instructions. Briefly, cells were transfected by a fast forward protocol. 100,000 cells/mL were transfected with a 1:1 mixture of HiPerFect reagent (1:200) and 100 nM of Cldn1-siRNA (Cldn1 siRNA_5 or Cldn1 siRNA_8) or negative control siRNA (control siRNA), respectively. Efficiency of KD was controlled by quantitative IHC of Cldn-1 intensity and by qRT-PCR analysis.

### Barrier function studies by TER assay

40,000 keratinocytes were plated on 24-well Transwell membrane filters (Corning Costar, Cambridge, MA) and cultured in DermaLife keratinocyte growth medium (Lifeline Cell Technology, Cellsystems, Troisdorf, Germany) containing 0.06 mM Ca^2+^. After cells reached confluency, medium was switched to medium containing different calcium concentrations (0.15–1.8 mM). For certain experiments cultures were kept under ALI conditions to improve differentiation^33^. TER was monitored daily using an EVOM epithelial voltohmmeter and electrode (World Precision Instruments Inc. New Haven, CT) according to the manufacturer’s instructions. In case of ALI conditions 0.5 mL of PBS was apically applied to the cultures to have a sufficient liquid level for the measurement which was removed after the measurements.

### Tracer permeability assays in cultured cells

Tracer permeability assays were performed as previously described^53^ with cells grown on culture plate inserts at the indicated time-points after Ca^2+^-switch. Briefly, 200 µL of 1.25 mg/mL FD4 (Sigma-Aldrich, Munich, Germany) were applied apically to the cell layers and incubated for 2 h at 37 °C. Subsequently the medium from the basal compartment was collected and fluorescence intensity was measured by using a fluorescence reader (Tecan, Maennedorf, Switzerland). The amount of tracer was calculated with a calibration curve. Tracer permeability was calculated as follows: Permeation coefficient Kp (cm/s) = flux (nmol⋅h^−1^⋅cm^−2^)/concentration (mmol/L) × 3.6. For *in vitro* inside-out barrier studies, transwell filters were placed basally in a 0.5 mg/mL solution of the 557-Da tracer EZ-Link sulfo-NHS-LC-biotin (Biotin-SH, Thermo Fisher Scientific, Darmstadt, Germany) and incubated for 45 min. Thereafter filters were fixed in 4% formaldehyde for 24 h, embedded in paraffin and 5 μm cross sections were prepared. Biotin-SH was analysed immunohistochemically as described below.

### Biotin-SH-barrier assay in human HFs

For TJ barrier investigation of human HFs, biopsies of 0.5 cm^2^ were taken from the donor scalp samples and prepared as previously described by^1,27^.

To study barrier functionality, biopsies were labelled with Biotin-SH. Briefly, 50 μl of 2 mg/mL Biotin-SH (557 Da) in PBS containing 1.0 mM CaCl_2_ were injected into the dermis and incubated for 2 h at 37 °C. After incubation, biopsies were fixed for 24 h in Formafix®4%, embedded in paraffin, and 5 μm longitudinal sections were cut using a Leica RM2165 Rotary Microtome (Leica Microsystems GmbH, Wetzlar, Germany). Paraffin sections were deparaffinated, rehydrated, and Biotin-SH was visualised by immunohistochemical staining (see below). For TJ opening experiments, HFs were freshly plucked from donor scalps and incubated for 30 min in PBS with and without EDTA (8 mM) prior to the Biotin-SH barrier assay.

### Immunohistochemical analysis of scalp tissue and cultured cells

Paraffin sections of human scalp biopsies and cells cultured on Transwell filters were derived as described above.

Antigen retrieval was achieved by microwave heating (2 × 10 min, 360 W in TEC buffer) prior to trypsin treatment (10 min, 0.001% trypsin at 37 °C). Thereafter sections were covered with DAKO “Serum-Free Protein Block”. Primary antibodies were diluted as summarized in Supplementary Table 1. Secondary antibodies were diluted as follows: Streptavidin-Texas Red against Biotin (1:600), Alexa Fab 488 (1:600) and Alexa Fab 594 (1:1250). An Axiophot II microscope (Zeiss, Goettingen, Germany) and the software Openlab 5.0.2 (Improvision, Coventry, UK) were used for the evaluation of the stainings. All images of stainings from a series of experiments were acquired and processed at the same settings, and representative areas were photographed. For immunohistochemical proof of apoptosis by the indirect TUNEL method, the ApopTag Fluorescein *In Situ* Apoptosis Detection Kit (Millipore, Darmstadt, Germany) was used according to the manufacturers manual for paraffin sections. Proliferative (Ki67-positive) cells were stained by MIB-1. For calculation of proportion of proliferative and apoptotic cells, MIB-positive and TUNEL-positive cells, respectively were counted. At least 3 visual fields (0.25 mm^2^) were evaluated per treatment (n = 3 different donors). In each visual field the numbers of Ki67 positive cells respectively TUNEL positive cells were normalized to the total numbers of cells which were evaluated by DAPI staining.

### Quantitative ROI based analysis

Quantification of TJ protein and Biotin-SH immunointensity was assessed by using Image J (version 2.0.0-rc-49/1.51d). Briefly, we determined the average fluorescence intensity of immunohistochemical stainings from at least 5 regions of interest (ROIs) with dimensions of 75 × 40 μm^2^ for TJ protein quantification and 75 × 25 μm^2^ for Biotin-SH quantification picked from the regions of specific staining of the target TJ protein or from the SC and SG for Biotin-SH in cell culture and CL and He* layer for Biotin-SH in HFs, and 2 ROIs from adjacent areas of non-specific background staining to measure the target-to-background ratio.

### qPCR

Total mRNA was isolated from cultured keratinocytes by using an RNeasy mini kit (Qiagen, Hilden, Germany) according to the manufacturer’s instructions. 50 ng of total RNA was used for first-strand cDNA synthesis with the cDNA synthesis kit (Thermo Fisher, Darmstadt, Germany) as suggested by the manufacturer. Four µL of the cDNA (1:25 diluted) was used as a template in real-time-PCR analysis with the FAM dye-labeled TaqMan MGB probes for Cldn-1 and GAPDH in an iCycler (Roche) under the conditions recommended by Applied Biosystems. All real-time-PCR analyses were performed in triplicate in three independent experiments. Relative transcriptional levels within distinct experiments were determined by using the 2^−ΔΔCt^ method^54^.

### Statistical analyses

Data are presented as means and SD or SEM. For statistical tests ANOVA was calculated using Excel 2010 (Microsoft, Redman, WA). ^*, §, $^p < 0.05; ^**, §§, $$^p < 0.01; ^***, §§§, $$$^p < 0.001.

### Data availability

The datasets generated and/or analysed during the current study are available from the corresponding author on reasonable request.

## Electronic supplementary material

Supplementary information
