# Supplementary material for: Tight Junction barriers in human hair follicles – role of claudin-1
Source: Sci Rep. 2018 Aug 24;8:12800. doi: 10.1038/s41598-018-30341-9 (PMC6109114; doi:10.1038/s41598-018-30341-9)
Supplement: Supplementary file 1 — Supplementary information [file 41598_2018_30341_MOESM1_ESM.pdf]

Supplementary information

Tight Junction barriers in human hair follicles – role of claudin-1

Michaela Zorn-Kruppa[1], Sabine Vidal-y-Sy[1], Pia Houdek[1], Ewa Wladykowski[1], Stephan Grzybowski[2], Robert Gruber[3], Christian Gorzelanny[1], Jason Harcup[4], Stefan W. Schneider[1] Amitabha Majumdar[5], Johanna M. Brandner[1*]

[1]Department of Dermatology and Venerology, University Hospital Hamburg-Eppendorf, Hamburg, Germany
[2]Medical One Klinik, Hamburg, Germany
[3]Department of Dermatology, Medical University of Innsbruck, Innsbruck, Austria
[4]Unilever R&D Port Sunlight Laboratory, Bebington, UK
[5]Unilever R&D Bangalore, Bangalore, India

[*] Corresponding author:

Johanna M. Brandner
Department of Dermatology and Venerology
University Hospital Hamburg-Eppendorf
Martinistrasse 52
20246 Hamburg
Tel: 0049-40-7410-55158
Fax: 0049-40-7410-58160
brandner@uke.de

Supplementary Figures

Supplementary Figure S1

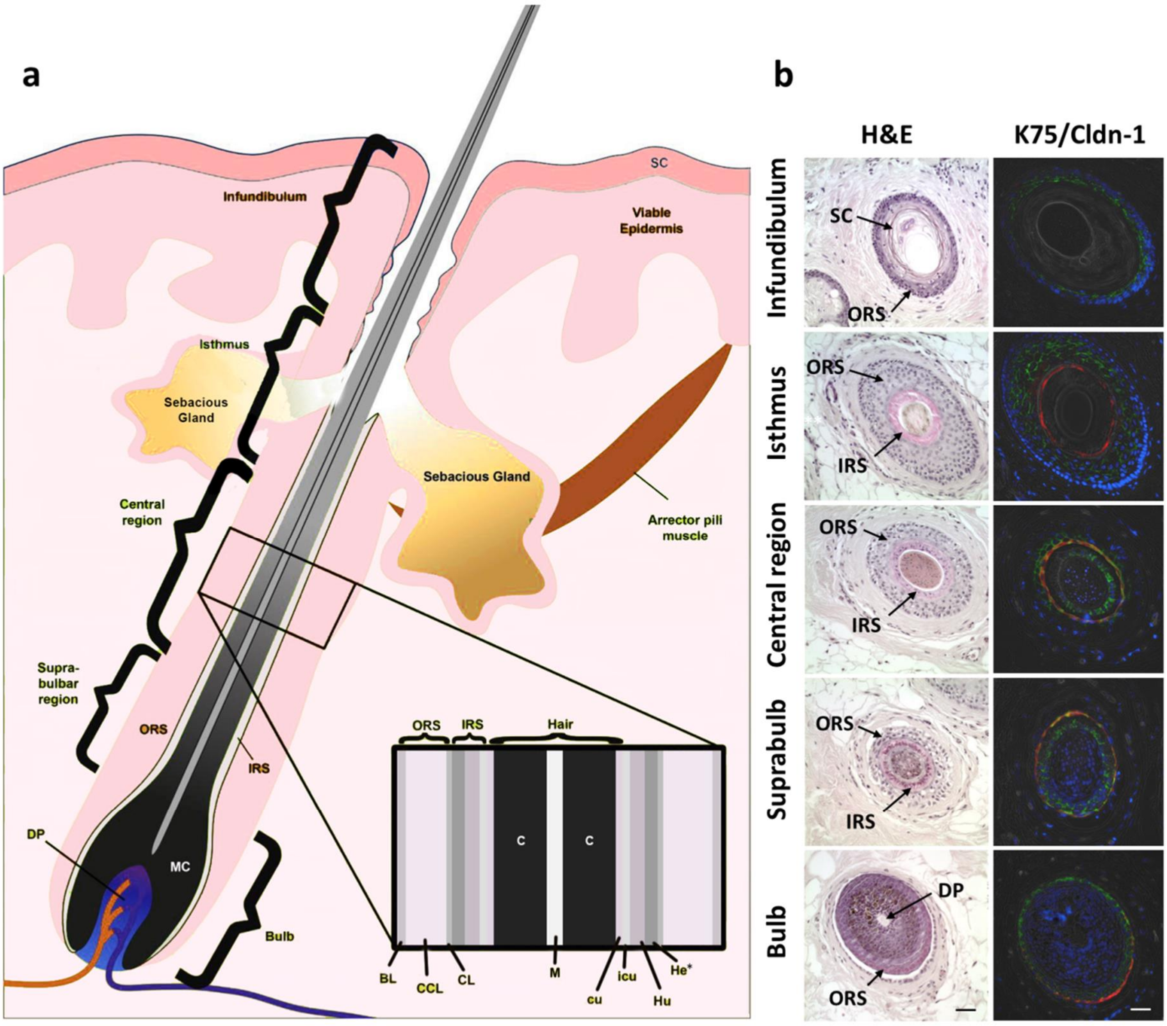

Summary of HF anatomy

(**a**) Scheme of a HF longitudinal section; BL = basal layer of ORS, C = cortex of hair, cu = cuticle of hair, CL = companion cell layer of ORS, CCL = central cell layer of ORS, DP = dermal papillae, He = Henle's layer of IRS (in the area of magnification already differentiated = He*), Hu = Huxley's layer of IRS, icu = cuticle of IRS, IRS = inner root sheath, M = medulla of hair, MC = matrix cells, ORS = outer root sheath, SC = stratum corneum. Modified from Mathes et al., 2016 (1). (**b**) Overall morphology and immunohistochemical characterization of cross sections from different segments of anagen HF showing H&E staining (left column) and immunohistochemical stainings of Cldn-1 (green) and K75 (red) (right column). Scale bar: 20 µm.

Supplementary Figure S2

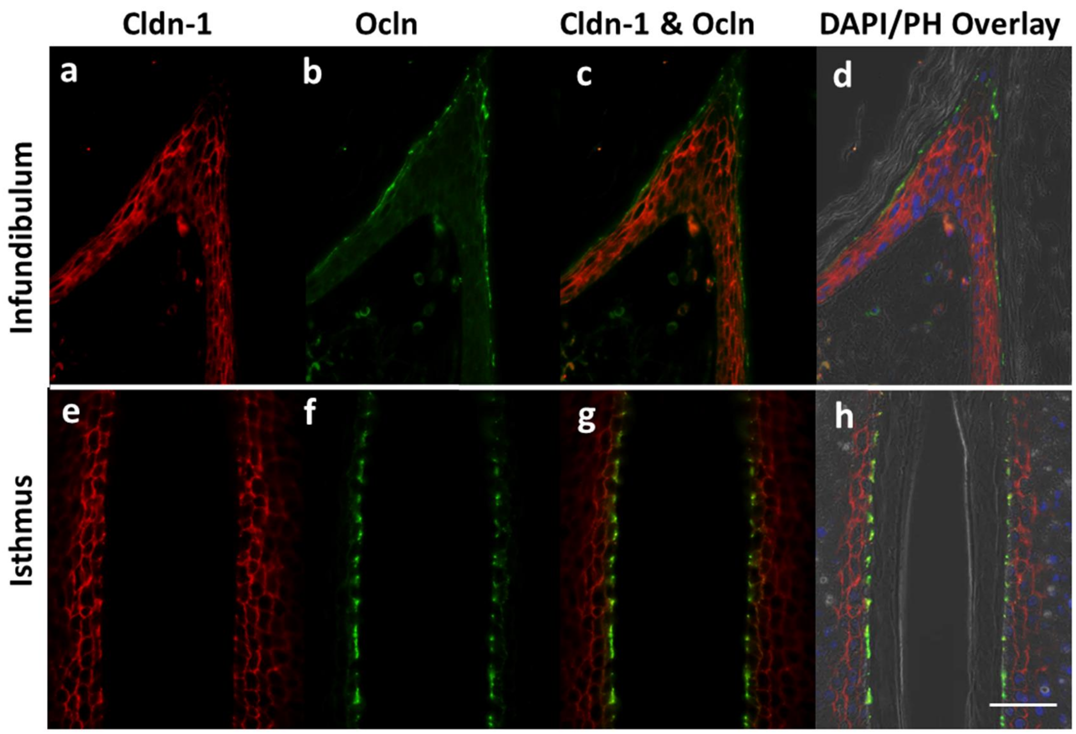

Immunolocalization of Cldn-1 and Ocln in infundibulum and isthmus of the anagen HF

Immunohistochemical stainings of Cldn-1 (red, **a,c,d,e,g,h**), and Ocln (green, **b,c,d,f,g,h**) in the infundibulum (**a-d**) and isthmus (**e-h**) regions of angen HFs. (**c,g**) overlay of Cldn-1 and Ocln staining. (**d,h**) Overlay of Cldn-1, Ocln, Dapi (blue nuclei) and phase contrast. Scale bar: 50 µm.

Supplementary Figure S3

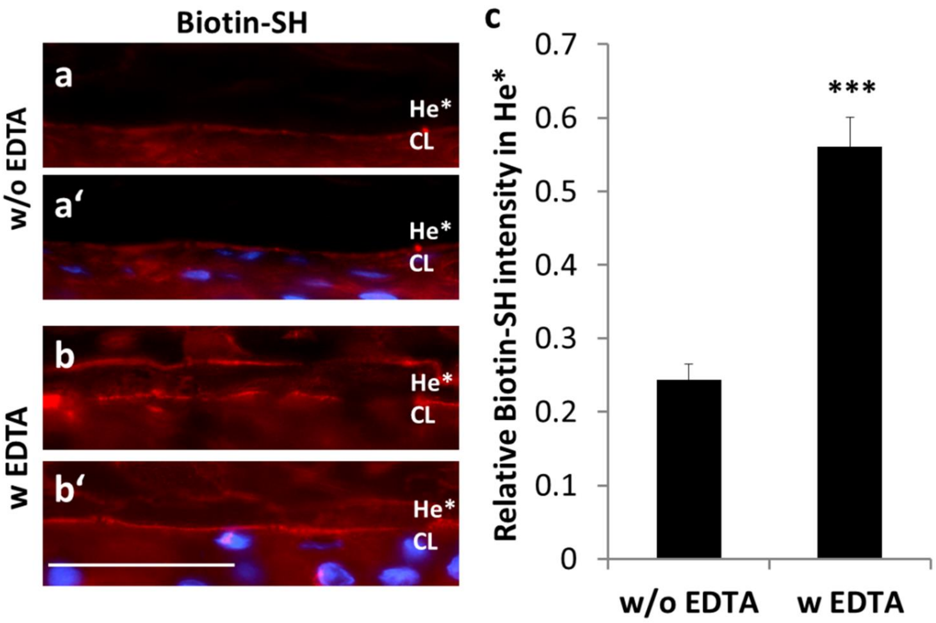

EDTA opens TJs in the hair follicle.

Biotin-SH permeation (red) in the central region of an untreated HF (w/o EDTA) with intact TJ barrier (**a, a’**), and with an impaired TJ barrier in the same region upon treatment of the tissue with 8.0 mM EDTA (w EDTA) (**b,b’**). (**a’,b’**) overlay images with DAPI staining (blue nuclei). CL: companion cell layer; He* differentiated Henle´s layer (**c**) Quantification of Biotin-SH intensities in He* layer of EDTA treated and untreated HFs relative to the intensity in the adjacent CL. Scale bar: 20 µm.

Supplementary Figure S4

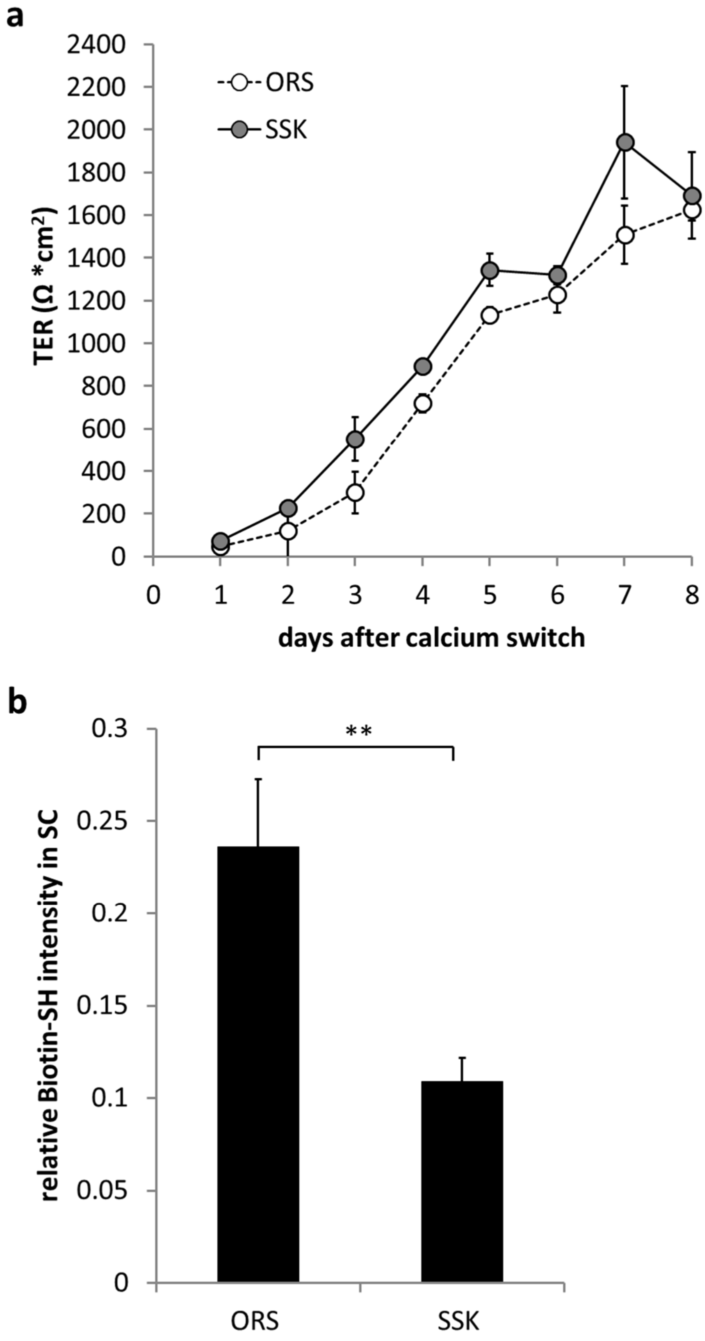

Barrier function in air-liquid interface cultures of ORS and SSK keratinocytes

(**a**) Representative time dependent TER curve of keratinocyte cultures isolated from ORS and scalp skin (SSK) and cultivated under air-liquid interphase (ALI) conditions. Means ± SD; triplicate wells *n* = 3 different donors; (**b**) Biotin-SH permeability into the SC of ALI cultures after basal application measured by Biotin intensity in SC relative to SG. Means ± SD; triplicate wells, *n* = 2 different donors; significant differences are indicated with *.

Supplementary Figure S5

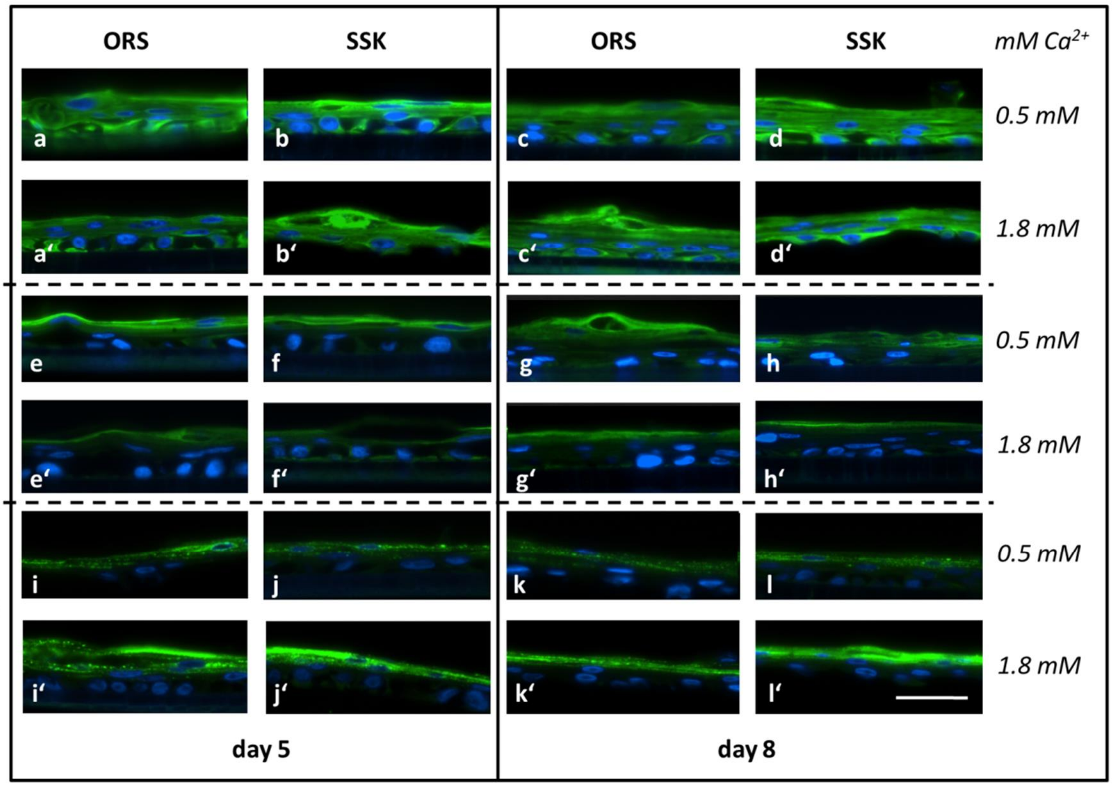

Characterization of submerged cultures of ORS and SSK cells regarding differentiation markers at different calcium concentrations

(**a-l,a'-l'**) Immunohistochemical stainings of differentiation markers Keratin 14 (K14; green, **a-d, a'-d'**), Keratin 75 (K75; green, **e-h,e'-h'**), and Filaggrin (Flg; green, **i-l,i´-l'**) in ORS (left) and SSK (right) cultures after 5 and 8 days of submerged cultivation. (**a-l**) 0.5 mM $CaCl_2$**;** (**a'-l'**) 1.8 mM $CaCl_2$. Scale bar: 20 µm.

Supplementary Figure S6

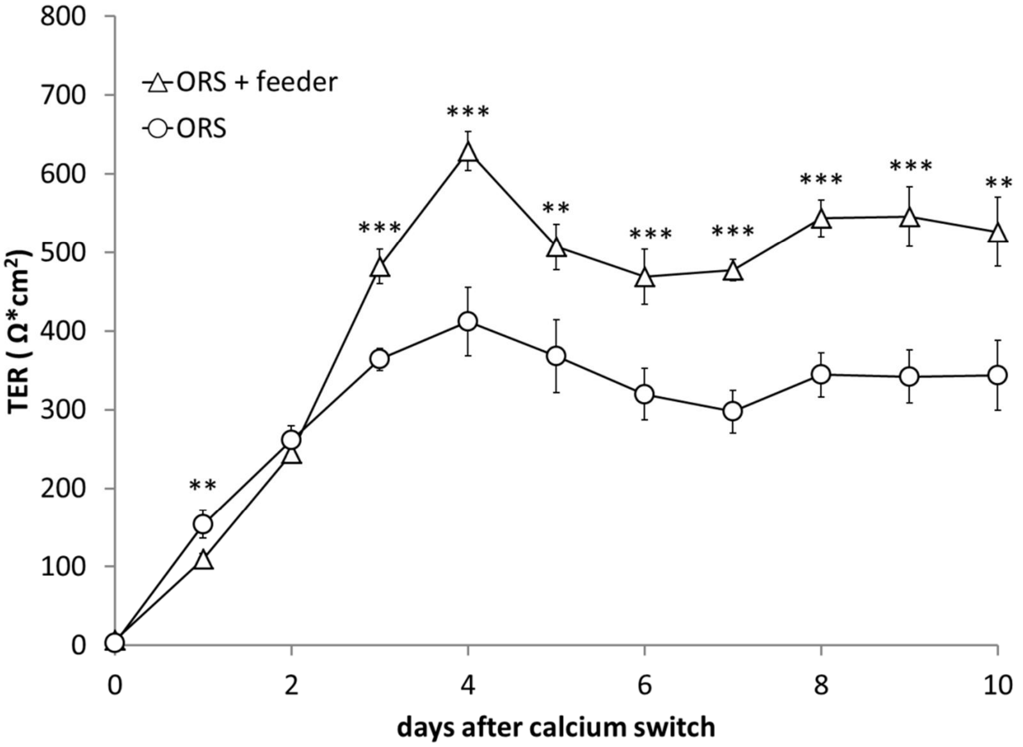

Influence of fibroblast feeder cultures on ORS keratinocytes

Representative time dependent TER curve of ORS keratinocytes cultured in presence or absence of fibroblast feeder cultures directly after isolation before first passage to serum-free medium. Means ± SD; triplicate wells, *n* = 3 different donors; significant differences are indicated with * (ANOVA).

Supplementary Figure S7

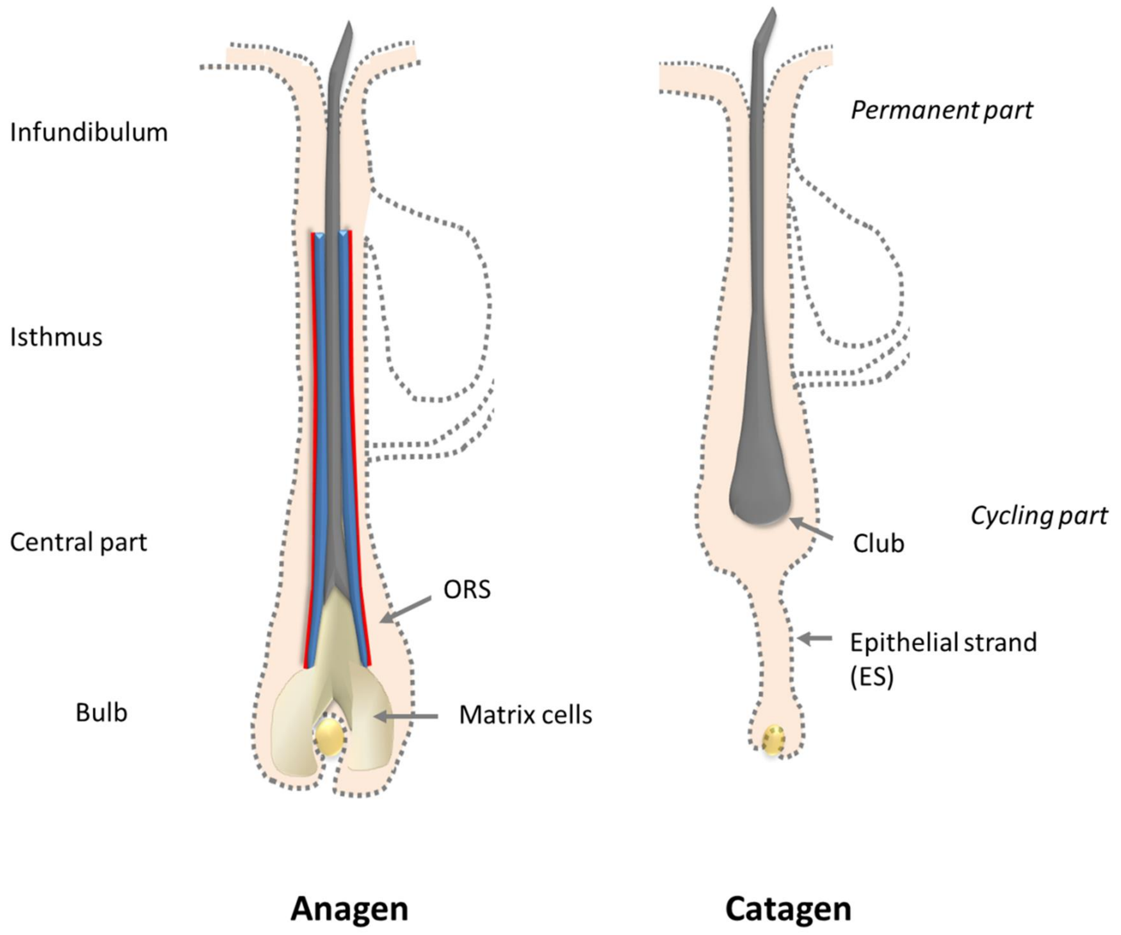

Schematic summary of anatomical changes during regression of a HF

ORS layers are marked in pale pink and matrix cells in beige. The companion cell layer is shown in red and IRS is tagged blue. The dermal papilla is shown in yellow.

Supplementary Figure S8

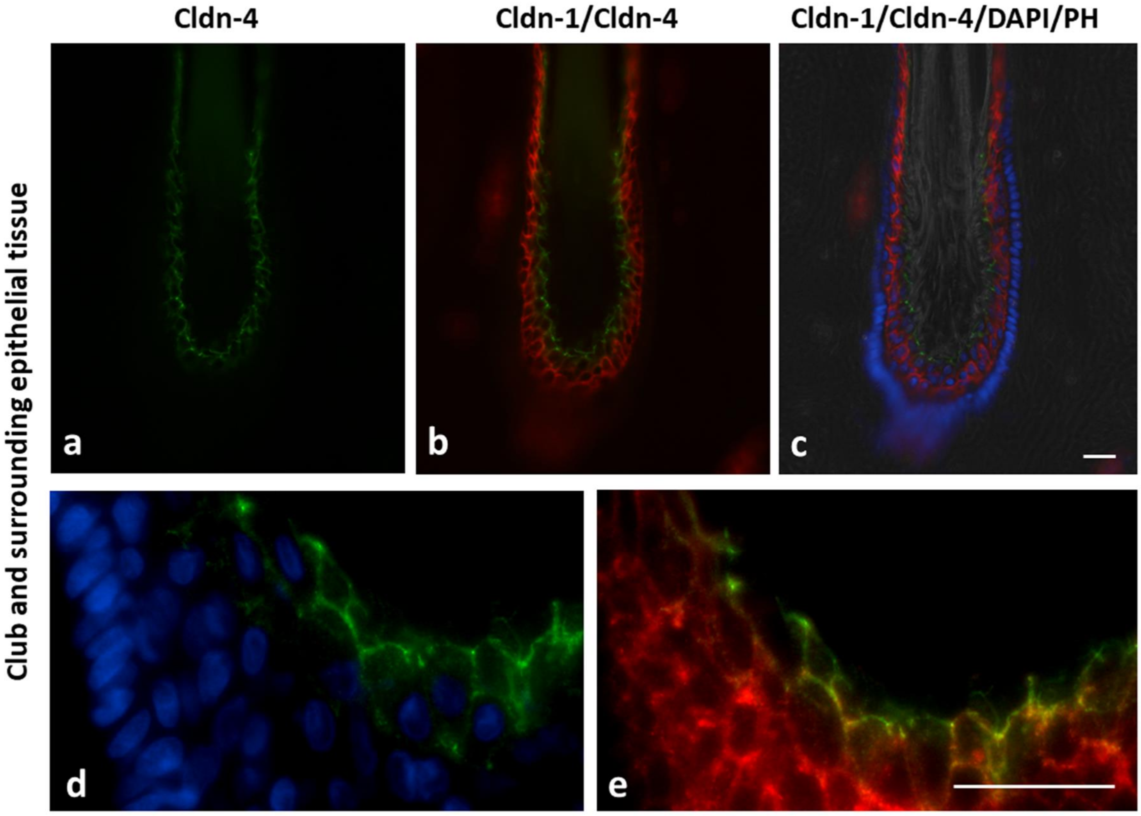

Immunolocalization of Cldn-4 and Cldn-1 in the club HF

Immunohistochemical stainings of Cldn-4 (green, **a,b,c,d,e**), and Cldn-1 (red, **b,c,e**) in the club HF. (**b**) Overlay of Cldn-1 and Cldn-4. (**c**) Overlay of Cldn-1 and Cldn-4 with DAPI staining (blue nuclei) and phase contrast. (**d,e**) magnifications of TJ protein localization in close proximity to keratinous rootlets of the club hair in a Cldn-4/DAPI overlay (**d**) and Cldn-1/Cldn-4 overlay (**e**). Scale bars: 20 µm.

Supplementary Figure S9

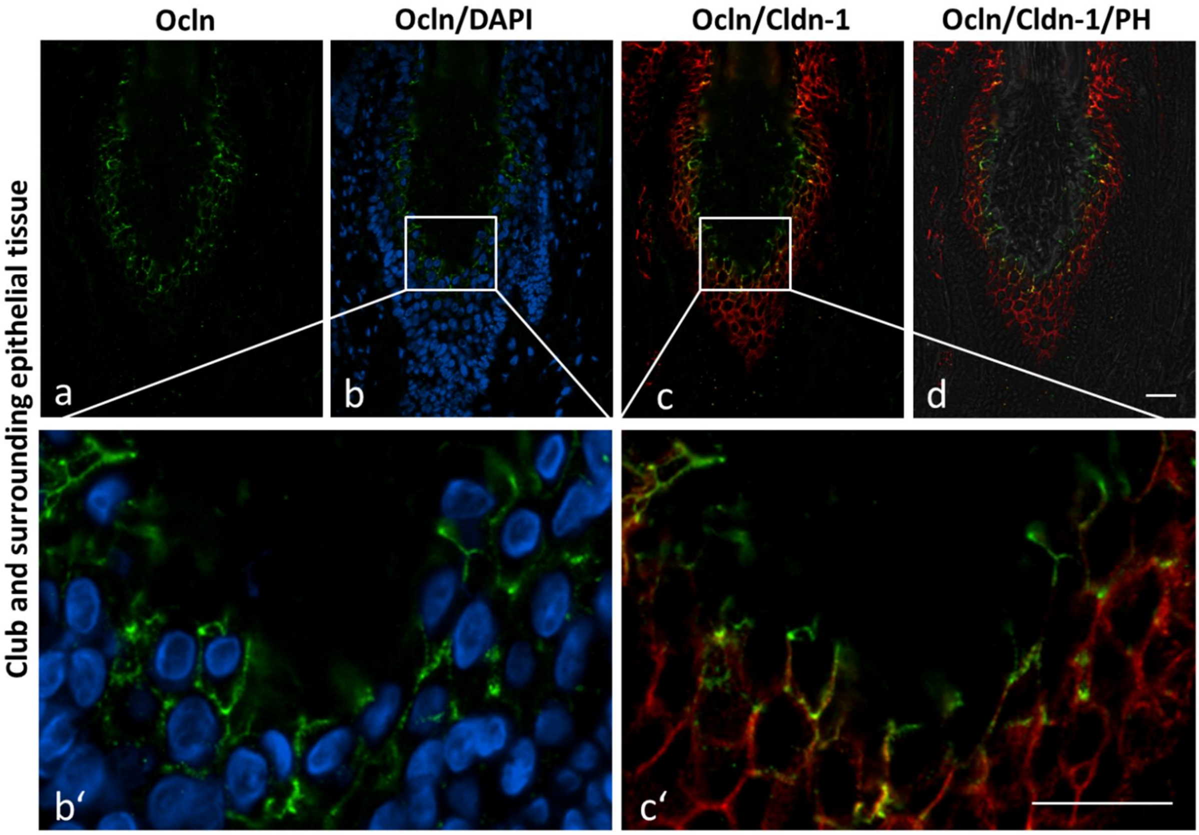

Immunolocalization of Ocln and Cldn-1 in the club HF

Immunohistochemical stainings of Ocln (green, **a-d,b',c'**), and Cldn-1 (red, **c,d,c'**) in the club HF together with DAPI staining (blue nuclei, **b,b'**) or phase contrast (**d**). (**b', c'**) magnifications of the areas in **b** and **c** showing TJ protein localization in close proximity to keratinous rootlets of the club. Scale bars: 20 µm.

Supplementary Figure S10

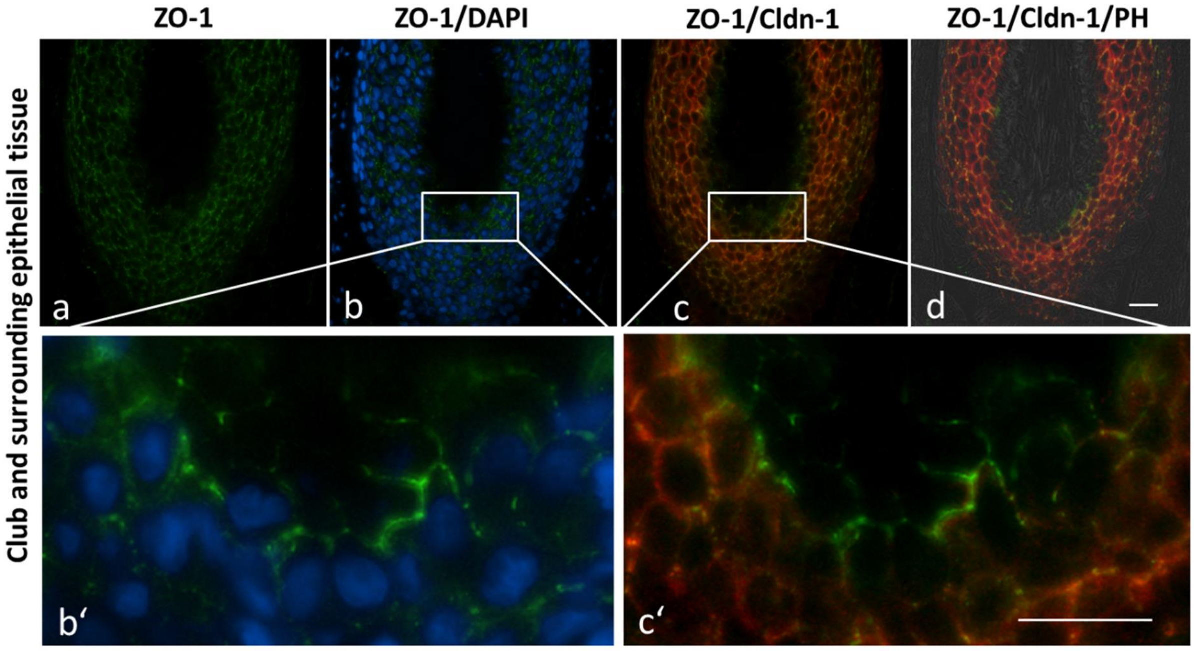

Immunolocalization of ZO-1 and Cldn-1 in the club HF

Immunohistochemical stainings of ZO-1 (green, **a-d,b',c'**), and Cldn-1 (red, **c,d,c'**) in the club HF together with DAPI staining (blue nuclei, **b,b'**) or phase contrast (**d**). (**b',c'**) magnifications of areas in **b** and **c** showing TJ protein localization in close proximity to keratinous rootlets of the club. Scale bars: 20 µm.

Supplementary Figure S11

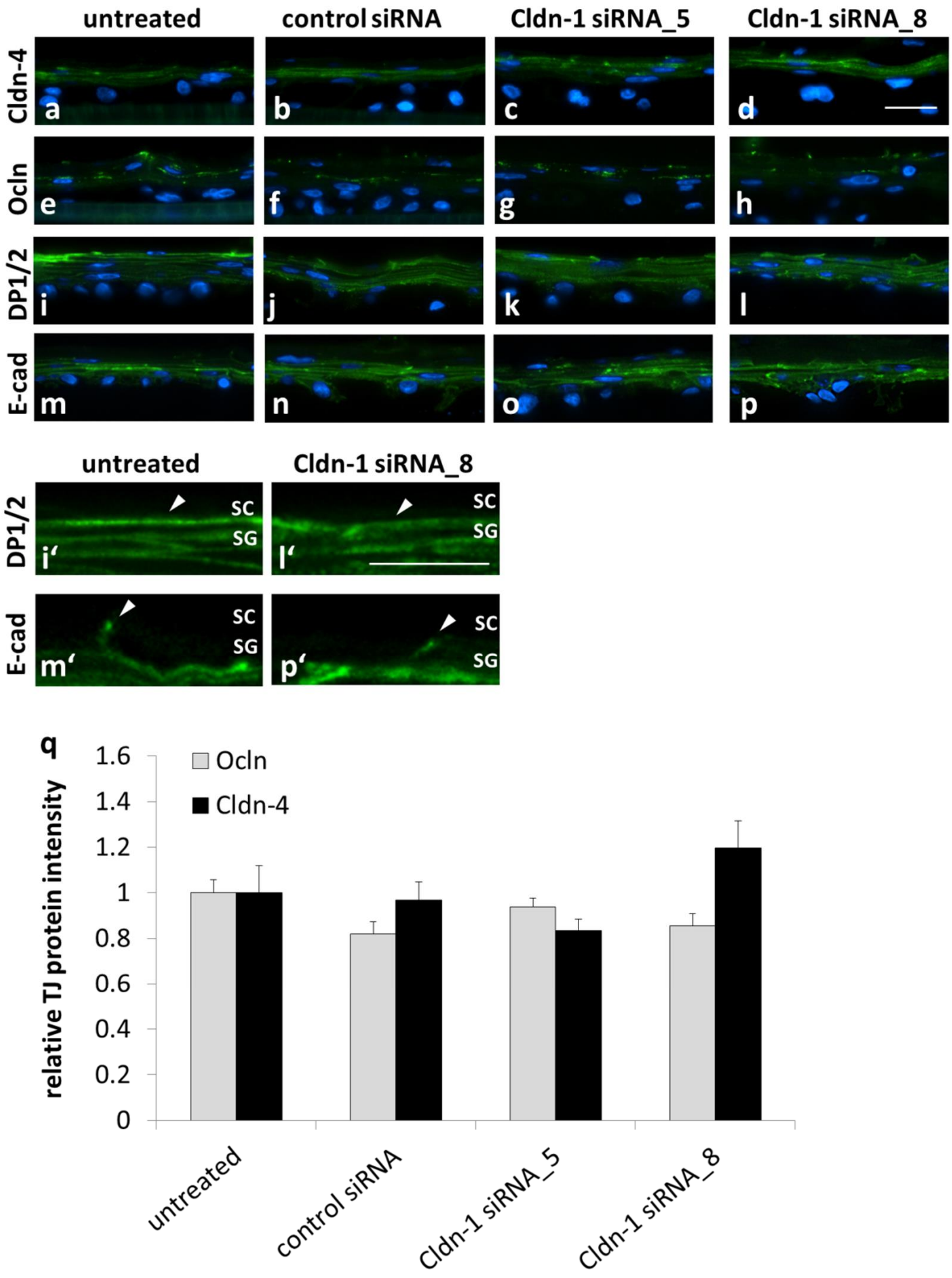

Characterization of Cldn1 knock-down cultures of ORS cells regarding several junctional proteins.

Immunohistochemical stainings of TJ proteins Cldn-4 (green, **a-d,**) and Ocln (green, **e-h**), desmosomal proteins Desmoplakins (DP) 1/2 (green, **i-l,i’,l’**) and adherence junction protein E-cadherin (green, **m-p,m’,p’**) in submerged cultured ORS keratinocytes after siRNA mediated KD of Cldn-1 using Cldn-1-siRNA_5 (**c,g,k,o**) and Cldn-1-siRNA_8 (**d,h,l,p,l’,p’**) in comparison to untreated cells (**a,e,i,m,i’,m’**) or negative control siRNA treated cells (**b,f,j,n**) at day 4. (**i’,l´,m’,p’**) magnifications of areas in **i,l,m,p** showing the polarized distribution of DP1/2 and E-cadherin in untreated and Cldn-1 siRNA_8 treated

cells. (**a-p**) Overlay of junctional protein and DAPI staining. Scale bars: 20 µm. (**q**) ROI based quantification of fluorescence intensity of Cldn-4 and Ocln in SG relative to unspecific background level.

Supplementary Tables

Supplementary Table S1

Antigen	Clone, Company	Species	Dilution for staining cells on Transwell filters	Dilution for staining scalp skin biopsies
Cldn-1	Clone: Jay 8, Thermo Fisher, Germany	rabbit	1:700	1:900
Cldn-4	Clone 3e2C1, Thermo Fisher, Germany	mouse	1:700	1:700
DP 1/2	Clones: DP1&2-2.15; DP1-2.17; DP1&2-2.20, Progen, Germany	mouse	1:5	1:5
E-cadherin	Clone: 5H9, Progen, Germany	mouse	1:5	1:5
Flg	NBP1-21310, Novus, Germany	rabbit	1:2500	
K14	Clone: LL002, Quartett, Germany	mouse	1:70	
K75	Provided by L. Langbein, DKFZ, Germany	guinea pig	1:3000	1:3000
Ki67	Clone: MIB-1, Dako, Denmark	mouse	1:30	
Ocln	N-19, Santa Cruz, Germany	goat	1:250	1:250
ZO-1	Clone: ZO-1-1A12, Thermo Fisher, Germany	mouse	1:200	1:200

List of antibodies used in the study

Antibodies and dilutions used for immunofluorescence stainings of cells on Transwell membrane filters and paraffin embedded HF biopsies.
